# Extracellular Vesicles as Precision Delivery Systems for Biopharmaceuticals: Innovations, Challenges, and Therapeutic Potential

**DOI:** 10.3390/pharmaceutics17050641

**Published:** 2025-05-12

**Authors:** Sidhesh Mohak, Zsolt Fabian

**Affiliations:** 1 Department of Medicine, South Texas Health System, McAllen, TX 78503, USA; smohak@mail.sjsm.org; 2 Department of Clinical Sciences, Saint James School of Medicine, Arnos Vale VC0280, Saint Vincent and the Grenadines; 3 School of Medicine and Dentistry, Faculty of Clinical and Biomedical Sciences, University of Central Lancashire, Fylde Rd, Preston PR1 2HE, UK; 4 Translocon Biotechnologies PLC, Akadémia u. 6, 1054 Budapest, Hungary

**Keywords:** biopharmaceuticals, extracellular vesicles, gene therapy, RNA therapeutics

## Abstract

Unlike traditional small-molecule agents, biopharmaceuticals, like synthetic RNAs, enzymes, and monoclonal antibodies, are highly vulnerable to environmental conditions. Preservation of their functional integrity necessitates advanced delivery methods. Being biocompatible, extracellular vesicles (EVs) gained attention as a promising system for delivering biopharmaceuticals, addressing challenges related to the stability and efficacy of sensitive therapeutic molecules. Indeed, EVs can cross biological barriers like the blood–brain barrier, delivering therapeutic cargo to tissues that are traditionally difficult to reach. Recent innovations in surface modification technologies, including ligand and antibody attachment, have further enhanced EVs’ targeting capabilities, making them particularly effective in personalized medicine. Here, we review the versatile suitability of EVs for being next-generation delivery vehicles of biopharmaceuticals, including current standings, practical challenges, and possible future directions of the technology.

## 1. Introduction

Personalized and precision medicine is fundamentally reshaping clinical practice by leveraging genetic information and molecular profiling to tailor complex health treatments [1,2,3]. This paradigm shift is particularly evident in the development and application of biopharmaceuticals, which are characterized by their larger and more intricate molecular structures compared to conventional, small-molecule drugs [4]. While traditional therapeutic agents weigh between 150 and 600 Da, biopharmaceuticals (e.g., monoclonal antibodies, proteins, and nucleic acid-based agents) exhibit a higher molecular weight and complexity, contributing to their unique mechanisms and therapeutic efficacy [5,6].

Unlike conventional small-molecule drugs that usually are taken orally, due to their stable nature in the acidic stomach, and allowed absorption through the intestinal epithelium, most biopharmaceuticals have to be administered through routes that bypass the gastrointestinal tract [7]. Indeed, high acidity and proteolytic enzymes in the gastrointestinal system, combined with their first-pass metabolism within the liver, compromise integrity and limit traditional oral delivery of these novel therapeutic agents [8,9]. Additionally, temperature sensitivity and the denaturation risk of biopharmaceuticals make them require careful formulation with excipients that preserve their structure and activity during storage and delivery [10,11].

These characteristics fuel the need for new delivery modalities that ensure the stability and efficacy of the biopharmaceuticals, providing protection from enzymatic degradation even in challenging environments, like the ocular surface, where enzymes like Cathepsin D, MMP-2, and MMP-9 can degrade biopharmaceuticals easily and rapidly [12,13,14,15]. The quest for novel drug delivery systems sheds light on lipid-based vesicular systems like the extracellular vesicles (EVs) [16]. Their capacity to encapsulate both hydrophilic and hydrophobic compounds and tunable release kinetics make them versatile carriers for various biopharmaceutical [17]. Moreover, by increasing drug permeation across biological barriers, EVs not only can enhance bioavailability but have unique potential in drug targeting to deliver biopharmaceuticals to specific cells or tissues [18].

## 2. Extracellular Vesicles

**Figure 1:**
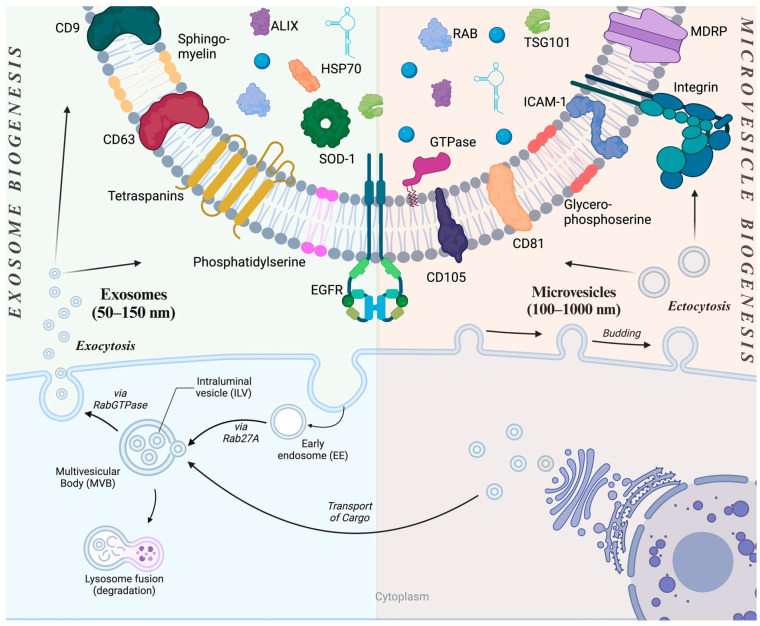
Comparative overview of exosome and microvesicle biogenesis and composition. Explored on the left in the figure, exosomes originate from the inward budding of endosomal membranes forming intraluminal vesicles (ILVs) within multi-vesicle bodies (MVBs). MVBs are destined for either lysosomal degradation or exocytosis via plasma membrane fusion. Exosomes are characterized by CD9, CD63, and CD81. In contrast, microvesicles (MVs, right side of the figure), are formed directly via the outward budding and fission of the plasma membrane. MVs are enriched in proteins like integrins and proteases, and lipids like phosphatidylserine. Shared components between the two types of EVs include nucleic acids (mRNAs, miRNAs, non-coding RNAs) and proteins involved in vesicle trafficking and fusion (e.g., ALIX, TSG101). EGFR: Epidermal Growth Factor Receptor; SOD-1: Superoxide Dismutase; HSP70: Heat Shock Protein-70; ALIX: ALG-2-interacting protein-X; RAB: Ras-associated binding protein; TSG101: Tumor susceptibility gene-101; ICAM-1: Intracellular adhesion molecule-1; MDRP: Multidrug Resistant Protein. Created with BioRender.

Extracellular vesicles are phospholipid bilayer-enclosed structures that are secreted from all cell types into the extracellular space, including biological fluids like plasma, breast milk, or saliva [19,20,21]. While several classes of extracellular vesicles exist, they are ultimately categorized based on their biogenesis, with exosomes and microvesicles being the two most prominent subtypes [22]. Exosomes are formed when newly endocytosed bodies (endosomes) are met with several intraluminal vesicles (ILVs) to form multivesicular bodies (MVBs), which then fuse with the plasma membrane for the exocytosis of the ILVs (Figure 1) [23,24]. The endosomal sorting complex required for transport (ESCRT) machinery plays a critical role in this process by sorting ubiquitinated proteins into ILVs [25,26,27]. Consequently, exosomes are considered “intracellular vesicles” while residing within MVBs, leading to the interchangeable use of the terms ILVs and exosomes. Exosomes are marked by CD9, CD63, and CD81, and carry selected miRNAs, siRNA, and heat shock proteins (HSPs) [28,29,30,31]. Conversely, microvesicles (MVs), which are also referred to as ectosomes, are formed through the direct outward budding of the plasma membrane, a process regulated by intracellular calcium levels and cytoskeletal remodeling (Figure 1) [32,33]. Their biogenesis also results in a molecular composition of their membrane that mirrors the plasma membrane composition of the cell of their origin [34]. In accordance, unlike exosomes, MV membranes are rich in integrins, proteases, and phosphatidylserine [34,35,36].

Both classes of EVs can carry a wide range of cargo molecules, either encapsulated or built in their lipid bilayer. These are nucleic acids, lipids, and proteins, including those involved in vesicle trafficking and membrane fusion, like ALIX and TSG101 (Figure 1) [30,32,37]. Deployed EVs can fuse with the cell membrane of the target cells or be internalized via endocytosis and deliver their cargo directly into recipient cells’ cytoplasm [38]. Accordingly, EVs loaded with mRNA can produce functional proteins in the recipient cell, indicating that cargo molecules, including labile mature messenger- or miRNAs, remain functional after delivery [39]. Moreover, the RNA cargo has been shown to be in a functional state even upon delivery via circulation to delicate distant target tissues like neurons in the central nervous system, demonstrating EVs’ capacity to protect labile cargo upon systemic administration [40,41].

Thus, EVs have emerged as a promising platform for precise drug delivery, offering several advantages over conventional, synthetic nanoparticles, like their safety profiles, unique biodistribution capabilities, and the ability to avoid recognition by the body’s defense systems, allowing them to remain in systemic circulation for extended periods [42,43,44].

## 3. Preparation of EVs for Therapeutic Purposes

### 3.1. Resourcing EVs

In order to obtain EVs for therapeutic purposes, including their use as drug delivery vehicles, both cellular and non-cellular sources can be used. Stem cell species like mesenchymal (MSC) or neural (NSC) ones are well known for their generous production of EVs naturally [45,46]. Because of their established clinical presence, low immunogenicity due to a lack of costimulatory marker-expression such as CD80 and CD86, and high EV-yields, MSCs are a leading source for EV-based drug delivery [47,48,49,50]. MSC-EVs also display stellar transfection efficacy, with one study showing that, following transfection with the tumor suppressive miR-146b in the GBM model, miR-146b levels increased roughly 7-fold in both MSCs and their exosomes compared to controls [51]. Their innate affinity for tumor and inflammatory tissues is facilitated by certain adhesion molecules expressed on their surfaces, such as CD44, that facilitate their preferential accumulation in injured/inflamed sites [52]. Indeed, human MSC-exosomes, when injected into mice with acute kidney injury, accumulated predominantly in the affected kidneys [53]. However, this preferential accumulation in areas of inflammation, such as the reticuloendothelial system, might not always be the desired outcome [54]. NSCs are also appealing for neuro-pharmaceutical applications because they generate EVs (NSC-EV) that exhibit innate CNS and blood–brain barrier (BBB) affinity via internalization by the BBB-endothelial cells through a heparan sulfate proteoglycan-mediated and dynamin-dependent endocytic pathway [55,56,57,58].

Besides the primary cell cultures, established cell lines like 293T are also suitable for the engineering and production of EVs [59,60]. In addition to being easily transfected, HEK293T-EVs display a comprehensive tissue distribution that stems from the diverse proteome they contain [61]. This vast distribution makes them useful in targeting and potentially fusing with various tissue membrane proteins, including those of B-cell lymphoma, lymph, eye, lung, bone marrow, and hepatocytes [61]. As a human embryonic kidney cell line, HEK293T cells naturally express MHC class I molecules such as HLA-A [62]. In antigen-specific applications, where engineered HLA-I molecules present peptides to activate CD8^+^ T cells, EVs derived from HEK293T can be beneficial [63]. However, in non-targeted EV therapies, allogeneic recipients may perceive mismatched HLA-I as foreign, which could result in immune clearance of EVs, CD8^+^ T-cell activation, and decreased therapeutic efficacy.

In addition, EVs can also be harvested from cell-free systems with unique properties that can be exploited in drug delivery. Indeed, milk-derived EVs, for instance, express typical exosome markers such as CD63 and CD81, and are absorbed via the neonatal Fc receptor, which stays active throughout life [64]. Cow milk-derived EVs have been shown to efficiently deliver engineered human miRNAs to human cells, suggesting their potential use for therapeutic purposes [63]. As naturally occurring components in milk, these EVs are generally regarded as safe, reducing concerns over immunogenicity or toxicity. However, the composition and function of milk-derived EVs are influenced by several factors like cow breed, diet, health, and lactation stage [65,66,67]. For instance, EVs produced from heat-stressed Brown Swiss cow milk improved cytoprotective responses by upregulating antioxidant and stress-response genes like *HMOX1*, *SOD1*, *CAT*, and *HSPA1A* that may affect the desired biological response of target cells [67].

Interestingly, EVs suitable to carry therapeutic cargo can be harnessed from plant-based systems as well. Plant-derived EVs are beneficial for pharmaceutical applications because they can be obtained from renewable resources like fruits, vegetables, and agricultural waste (like juice pulp or peel waste). Moreover, they are proving to be a promising candidate in overcoming multidrug drug resistance. Indeed, through caveolin-mediated endocytosis, macropinocytosis, and clathrin-mediated endocytosis, heparin-modified lemon-derived EVs loaded with doxorubicin showed an increased uptake in doxorubicin-resistant cancer cells [68]. However, compared to mammalian EVs, their surface markers and mechanisms of action are less defined due to lack of research.

### 3.2. EV Yield Optimization

Exosome secretion can be altered via genetic engineering techniques that target the genes associated with EV biogenesis and release, such as TSG101, ALIX, CD63, CD9, Rab family, etc. [69]. Indeed, researchers designed an exosome-to-cell device to produce a high yield of EVs from cells. More specifically, they found that co-expression of ‘synthetic EV-production boosters’ such as STEAP3, syndecan-4, and L-aspartate oxidase fragment resulted in a 40-fold increase in EV production without affecting exosome size [70]. Another way to alter EV yield is via placing the cells of choice under hypoxic conditions. In a study of the myocardial infarct model, hypoxia-treated MSC-Exo led to a better amelioration of myocardial infarction compared to the untreated group, and these changes were characterized by increased vascular density, decreased myocardial apoptosis, and reduced cardiac fibrosis [71]. Hypoxia inducible factor (HIF) is a family of transcription factors that are synthesized in normoxia but swiftly degraded by VHL-E3 ligase protein [72]. RAB27a and RAB27b are key proteins involved in exosome release, as they translocate MVBs to the cell membrane, leading to their fusion [73]. It has been illustrated that *HIF1-α* expression is also linked with peak expression of exosome proteins such as Rab27a, Rab27b, ALIX, and TSG101. This suggests that hypoxia-induced expression of *HIF1-α* is associated with exosome biogenesis [74]. RAB7 is a protein involved in exosome transport to lysosomes, resulting in their degradation. Interestingly, hypoxia has also been shown to activate STAT3, a protein that downregulates RAB7 while upregulating RAB27a, resulting in exosomes avoiding lysosomal degradation and favoring membrane fusion followed by extracellular release [75]. Pretreatment with cytokines is also an avenue that is being employed to further increase EV yield and alter exosome cargoes. Indeed, IL-β pretreatment was shown to upregulate miR-146a expression in BMSC-Exo [76]. These cytokine-treated exosomes exhibited anti-inflammatory effects in osteoarthritis SW982 cells, an effect mediated by miR147b, along with the inhibition of the NF-kB pathway [77]. Another study also found that pretreatment of gingival MSC-Exos with TNF-*α* led to increased CD73 expression, a common MSC marker. Interestingly, this effect was MSC-specific, as the levels of CD73 mRNA were unchanged in endothelial cells and astrocytes, suggesting that cytokine pretreatment has the capability of not only targeting exosome production but also cell-specific yields [78].

Several methods exist to isolate EVs from either cell culture media or other fluids (Table 1). Of the various methods used in modern EV research, the four most common are Ultracentrifugation (UC), Extrusion, Ultrafiltration (UF), and Size Exclusion Chromatography (SEC). Ultracentrifugation is the most commonly used method for EV purification. To efficiently remove cell debris and other foreign materials and produce EVs, this traditional approach employs successive high-speed centrifugations [79]. However, UC is limited by EV aggregation and severe shear forces that may reduce the yield [80,81]. Extrusion is a method that uses nanoporous membranes to extrude cells, yielding nanovesicles [82]. This technique uses lipid bilayer shuffling and friction forces to create cell-derived nanovesicles in a matter of seconds [83]. Ultrafiltration separates substances based on their molecular weight, making it ideal for high-throughput isolation methods from large volume samples. However, particles similar to EVs in size can also penetrate membranes and “clog” the membrane, leading to lower EV recovery rates. Thus, UC and UF are typically combined to isolate EVs with a higher purity yield [84]. Lastly, size exclusion chromatography separates EVs according to their size [85]. Limitations of SEC include dilution of the EV sample, although it is outweighed by its comparatively quick processing durations and ability to preserve vesicle properties after elution [86,87].

### 3.3. EV Characterization Techniques

For exosomes to be safe and suitable for mass production, their characterization is a crucial step that is required before clinical translation (Table 1). Exosome characterization can broadly be classified into two categories: qualitative and quantitative. Quantitative analysis assesses measurable parameters such as particle size, concentration, and cargo abundance, while qualitative analysis gauges exosome morphology, surface marker identity, structural organization and chemical composition. One of the most important measures in this process is particle number and size determination, commonly performed by nanoparticle tracking analysis (NTA). NTA assesses vesicle concentration and size distribution by Brownian motion and provides a reasonable estimate of the yield and formulation uniformity [88]. This is complemented by protein and lipid profiling to describe the cargo composition.

For molecular characterizations, mass spectrometry provides label-free, high-resolution profiling of protein and lipid content, whereas ELISA is a specific counter for the detection of single surface or cargo proteins, particularly when antibodies are of good quality [89,90]. These two methods together yield information on the purity and identity of EV populations [91]. In addition to lipids and proteins, exosomes also carry functional RNA and DNA species. To identify these, next-generation sequencing (NGS) offers high-throughput transcriptome analysis in thousands of RNA species, whereas polymerase chain reaction (PCR) remains the gold standard for the identification of known sequences with high specificity and sensitivity [92,93]. Apart from molecular characterization, it is likewise significant that the chemical composition and biophysical features of exosomes are identified. Raman spectroscopy, as a non-destructive, label-free technique, supports chemical fingerprinting through vibrational bond analysis that discriminates EVs from non-vesicular contaminants [94].

**Figure 2:**
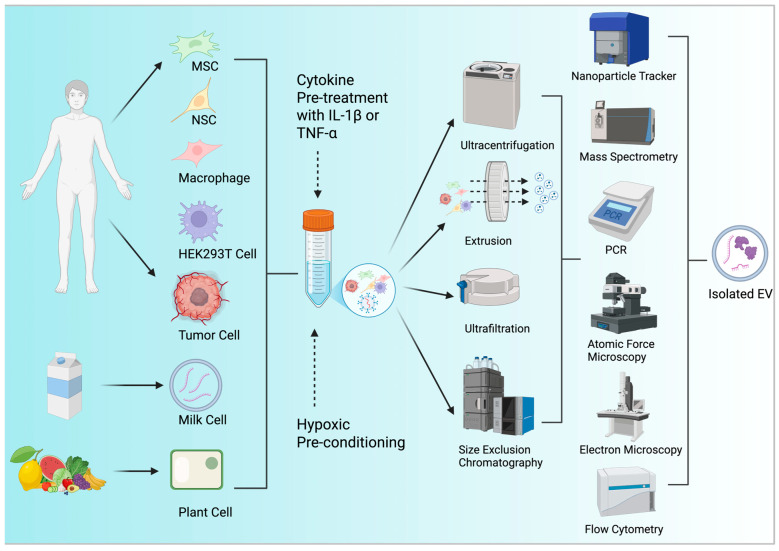
Extracellular vesicle sources, yield optimization, isolation, and characterization strategies. Extracellular vesicles can be derived from mesenchymal stem cells (MSCs), neural stem cells (NSCs), macrophages, tumor cells, HEK293T cells, milk cells, and plant-derived cells (e.g., lemon EVs). These EVs can be obtained from body fluids such as saliva, urine, blood, cerebrospinal fluid (CSF), and lymph, or directly from milk and plants. To enhance EV yield prior to isolation, donor cells can undergo yield optimization strategies such as hypoxic preconditioning, cytokine stimulation (e.g., TNF-α, IL-1β), or genetic engineering targeting key EV biogenesis regulators (e.g., Rab27a/b, TSG101, ALIX, CD63). To isolate EVs, most implemented techniques include ultracentrifugation (high-speed spinning to separate EVs based on density), extrusion (forcing fluids through nanoporous membranes for size-based separation), ultrafiltration (filtering EVs through membranes of specific pore sizes), and size-exclusion chromatography (separating EVs from contaminants based on molecular size differences). Once isolated, EVs are characterized using methods such as nanoparticle tracking analysis (NTA) for size/concentration, mass spectrometry for cargo profiling, PCR for nucleic acid quantification, atomic force microscopy (AFM) and electron microscopy (EM) for morphology, and flow cytometry for surface marker profiling. The result is a purified EV population ready for use. Created with BioRender.

To establish the exosomal surface architecture and spatial organization of the component surface, atomic force microscopy (AFM) offers 3D high-resolution mapping in conjunction with information on mechanical properties such as stiffness and adhesion [95]. At the same time, scanning electron microscopy (SEM) can provide high-resolution surface imaging, albeit with the requirement of sophisticated sample preparation that is potentially disruptive of native morphology [96]. For exact size measurement, electron microscopy remains unrivaled in single-vesicle-size resolution with high precision, although low throughput [97]. Flow cytometry, further increasingly optimized for nanoparticle detection, enables fast multiparametric analysis of surface markers to aid phenotypic profiling of heterogeneous EV populations (Figure 2) [98].

**Table 1:** Summary of the most commonly used EV research tools and platforms, including isolation and characterization methods. Each entry details the method of use, its key advantages, and potential disadvantages in EV-based biopharmaceutical delivery.

EV Processing Stage	Method	Advantages	Disadvantages	Reference
EV Isolation	Ultracentrifugation (UC)	▪ Widely used and accepted in EV research ▪ Capable of separating EVs from large sample volumes	▪ Shear forces can damage EVs ▪ Aggregation may reduce purity ▪ Time-consuming and equipment-intensive	[79,80,81]
	Extrusion	▪ Rapid vesicle production ▪ Mimics natural vesicle size via mechanical extrusion	▪ May disrupt vesicle integrity ▪ Less selective than other methods	[82,83]
	Ultrafiltration (UF)	▪ High throughput ▪ Useful for processing large volumes ▪ Size-based separation	▪ Membrane clogging reduces recovery ▪ Co-isolation of similarly sized particles	[84]
	Size Exclusion Chromatography (SEC)	▪ Preserves EV structure ▪ Fast processing time ▪ Useful for downstream applications	▪ Sample dilution ▪ Lower yield than UC in some cases	[85,86,87]
EV Characterization by Total Number of Exosomes	Nanoparticle Tracking Analysis (NTA)	▪ Provides both particle count and size distribution ▪ Widely used in EV research ▪ Direct visualization of vesicle motion	▪ Affected by sample heterogeneity ▪ Limited resolution for very small vesicles	[99,100]
EV Characterization by Surface Markers and Protein Numbers	Mass Spectrometry	▪ High-resolution, label-free protein identification ▪ Enables broad proteomic profiling	▪ Requires complex sample preparation and instrumentation ▪ Not suitable for live tracking	[101]
	ELISA	▪ Highly specific ▪ Widely accessible ▪ Useful for known target proteins	▪ Limited to pre-selected markers ▪ Dependent on antibody quality	[90]
EV Characterization by Lipid Content	Raman Spectroscopy	▪ Label-free ▪ Provides molecular bond-level lipid profiling	▪ Low throughput and sensitivity ▪ Requires expensive equipment	[102]
EV Characterization by DNA/RNA Content	Next Generation Sequencing (NGS)	▪ Comprehensive profiling ▪ Detects novel sequences ▪ High accuracy	▪ Costly ▪ Requires computational analysis	[93]
	Polymerase Chain Reaction (PCR)	▪ Highly sensitive and specific for known targets ▪ Gold standard for quantification	▪ Requires prior sequence information ▪ Prone to contamination	[93]
EV Characterization by Structure	Atomic Force Microscopy (AFM)	▪ Provides high-resolution 3D imaging ▪ Measures mechanical properties	▪ Slow scan speed ▪ Limited to small sample areas	[95]
	Scanning Electron Microscopy (SEM)	▪ Excellent surface detail ▪ Large depth of field	▪ Requires dehydration and vacuum ▪ May distort native structure	[103]
EV Characterization by Size	Flow Cytometry	▪ High throughput ▪ Can analyze surface markers simultaneously	▪ Size overestimation due to swarming ▪ Lower sensitivity for small EVs	[104]
	Electron Microscopy	▪ Most accurate size determination of single vesicles	▪ Low throughput ▪ Requires complex sample preparation	[105]
EV Characterization by Chemical Composition	Raman Spectroscopy	▪ Label-free and non-destructive ▪ Reveals molecular bond-level composition	▪ Low sensitivity ▪ Limited throughput	[102]
EV Characterization by Topology	Atomic Force Microscopy	▪ High-resolution 3D mapping ▪ Functional force measurements	▪ Requires surface immobilization ▪ Slow imaging	[95]

### 3.4. EV Cargo Loading Strategies

**Figure 3:**
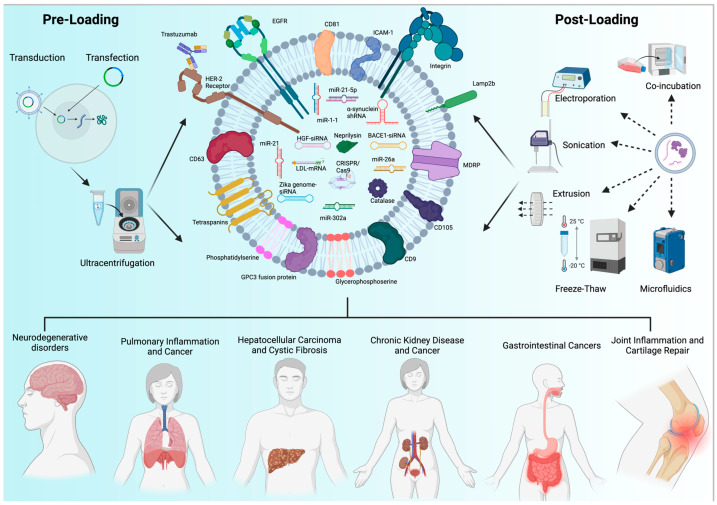
Cargo loading strategies for extracellular vesicle-mediated biopharmaceutical delivery. Pre-loading includes the natural integration of cargo into EVs during biogenesis, as well as the engineering of donor cells by transduction and transfection. Co-incubation, electroporation, sonication, extrusion, freeze–thaw, and microfluidics are post-loading techniques that include introducing cargo directly into isolated EVs. These techniques enable the loading of a variety of therapeutic molecules, such as membrane proteins (Lamp2b, Glypican-3 (GPC3) fusion protein, E7-Lamp2b fusion), siRNAs (HGF-specific siRNA, *BACE1* siRNA, and Zika virus genome-specific siRNA), mRNAs (*LDLR* mRNA), CRISPR/Cas9 constructs, and enzymes/proteins (Neprilysin/CD10, Catalase, and monoclonal antibodies like Trastuzumab). Neurodegenerative diseases, lung cancer, hepatocellular carcinoma, cystic fibrosis, kidney cancer, gastrointestinal cancers, and inflammatory diseases like osteoarthritis may all benefit from the use of these EV-based treatments, which can be delivered intradermally, intranasally, intramuscularly, or subcutaneously. Created with BioRender.

Using EVs as delivery vehicles for therapeutically active compounds, resourced, isolated, and characterized EVs need to be loaded with the desired cargo. There are two major approaches for cargo loading, depending on the timing of cargo accumulation and EV genesis (Figure 3). Pre-loading refers to the EV loading process that relies on the cellular uptake of the cargo molecules by the donor cells preceding the formation of EVs loaded with therapeutic agents [106]. This approach leverages natural processes but often requires optimization to enhance cargo encapsulation efficiency [107,108,109].

For complex biopharmaceuticals, like miRNAs and siRNAs, transfection of the donor cells has been demonstrated as a reliable technique for cargo loading of therapeutic EV genesis. Both transient and stable transfections have been reported to be used successfully to enrich the desired RNA cargo in EVs. Cargo loading can be facilitated using ultrasound combined with microbubbles technology that enhances drug loading by stimulating vesicle release, although the technique risks degradation of sensitive cargo in endosomal pathways [110,111]. Via transient transfection, miRNAs or their sponge constructs, like miR-1 and miR-21-5p, that are altered in pathologies such as glioblastoma, were enriched in EVs for therapeutic purposes [112,113]. Using a sponge construct of miR-21 in HEK293T cells, stable expression resulted in miR-21-sponge-loaded EVs that were successfully used for suppressing miR-21 activity in a glioblastoma rat model [114]. If they need to be derived from donor cells, like stem cells, that are naturally challenging to transfect, lentiviral-mediated overexpression of RNA species, like miRNAs and siRNAs, has been shown as an efficient method to encapsulate regulatory RNA molecules in EVs [115,116]. Combination of simple incubation-based drug uptake with lentivirus-mediated transduction has also been shown as a feasible method to load EVs with chemically different cargos. Indeed, lentiviral-transduced MSCs expressing human TRAIL incubated in the presence of cabazitaxel resulted in the production of MSC-derived exosomes loaded with both cabazitaxel and TRAIL that demonstrated potent therapeutic activity in an oral squamous cell carcinoma model [117].

In contrast to pre-loading, post-loading strategies refer to the direct loading of EVs with various therapeutic substances. Incubation of isolated EVs with various drugs under controlled conditions shows comparable cellular uptake and therapeutic activity to that of their pre-loaded counterparts while allowing for more precise control over drug incorporation into isolated EVs [118]. These techniques can be of particular use in terms of customizing EVs with antibody conjugation or peptide incorporation for specific therapeutic applications but may impact vesicle integrity [119,120]. Although for conventional therapeutic compounds, co-incubation of purified EVs is the most commonly used method for post-loading; complex biopharmaceuticals require more active manipulations of EVs upon post-loading [121]. One of these is electroporation that uses electric pulses to introduce cargo into EVs and that enables the automated high-throughput workflow of encapsulation, simplifying drug loading of purified EVs [122]. This method is suitable for loading nucleic acid cargo due to its hydrophilic nature and typically generates a great amount of drug-loaded EVs [123]. Sonication of isolated EVs is another technique that allows for cargo post-loading via sound waves that disrupt vesicle membranes. This method yields a high loading efficiency and has been demonstrated to be suitable for larger hydrophilic cargo like siRNAs and proteins alike [124,125,126]. Extrusion is a physical loading technique for isolated EVs, in which cargo is mixed and encapsulated by forcing EVs and cargo via nanoscale holes. Despite concerns, it has been demonstrated that the technique preserves the loaded cargo’s biological activity without causing appreciable damage [127].

Another post-loading method is the freeze–thaw approach that entails incubating EVs with cargo before repeatedly freezing and thawing them to break and re-form their membranes; but low reported loading efficiency and the potential damage of sensitive biopharmaceuticals seem to limit the use of this strategy for post-loading of complex therapeutic agents [125]. A potential bypass of these limitations is the combination of EV extraction techniques, as seen in a glioblastoma multiforme model, where the freeze–thaw method was used in combination with sonication and co-incubation to create dual receptor-specific exosomes loaded with temozolomide and benzylguanine [128]. The general disadvantages of post-loading strategies, however, is that they may compromise vesicle integrity and flexibility. This can be avoided using microfluidics and acoustofluidics systems that combine ultrasound, microchannel, and acoustic waves to increase loading efficiency while maintaining EV integrity [129,130].

### 3.5. EV Surface Modifications

The lipid bilayer of EVs is not only suitable for encapsulating and protecting the cargo from the harsh surrounding environment but may ensure biocompatibility with biological interfaces. Despite being biocompatible and having natural homing properties, however, EVs may have limited endogenous targeting specificity and, thus, can accumulate in non-target organs like the liver and spleen [131]. Surface modifications can enhance their therapeutic potential by improving delivery efficiency via enhanced targeting and, thus, reduced circulation time [132]. These can either be endogenous (cellular-level engineering) or exogenous (post-isolation changes).

Endogenous surface modifications use genetic or biochemical techniques to produce EVs with desired surface markers directly from donor cells. Indeed, lentiviral transduced cells have been successfully used to produce C-X-C motif chemokine receptor 4-coated exosomes that, with the desired miRNA cargo, showed enhanced anti-inflammatory effects via macrophage targeting [133]. Copper-free click chemistry is another aspect of cellular level engineering that enables precise EV modifications without interfering with its function. By connecting azide groups with strained cyclooctyne derivatives, such as DBCO, strain-promoted azide-alkyne cycloaddition (SPAAC) allows for targeted surface modification without the need for copper catalysts [134]. This prevents copper-induced oxidative stress and membrane damage, maintains EV integrity, and facilitates the effective attachment of therapeutic or targeting molecules to the membrane of EVs [134]. Azide-labeled exosomes, for instance, are successfully fluorescently tagged for real-time tracking [135].

In contrast, exogenous modifications directly alter isolated EV membranes, allowing the integration of functional molecules. Techniques that temporarily disrupt lipid membranes of EVs, like sonication, extrusion, or freeze–thaw, allow insertion of targeting molecules into the lipid bilayer of the vesicles [136]. Covalent bonds or hydrophobic interactions are being used to attach functional groups to the surfaces of EVs. Using this approach, lipid tail-modified targeting peptides or fluorescent labels have been successfully anchored to EV membranes [99,137].

## 4. EVs as Delivery Vehicles for Biopharmaceuticals

Biopharmaceutical innovations like synthetic nucleic acids or proteins with designed functions have opened treatment options for previously untreatable clinical conditions [138,139,140,141]. These new medications, however, pose challenges in terms of their delivery to target tissues, as non-specific distribution or immune recognition significantly limit their therapeutic effect [142,143,144]. To bypass these issues, EVs have been proposed as delivery vehicles for biopharmaceuticals due to their biocompatibility, negligible immunogenicity, and wide range of potential cargo (Table 2).

Indeed, genetically altered MSCs expressing a fusion protein of the exosome membrane protein Lamp2b and a single-chain variable fragment (scFv) specific to the hepatocellular carcinoma (HCC) marker Glypican-3 (GPC3) were successfully used to specifically deliver the biopharmaceutical cargo miR-26a to GPC3-positive cancer cells both in vitro and in vivo, which resulted in cell cycle arrest via downregulation of cyclin D2 and E2, respectively, and, consequently, inhibited tumor cell proliferation [145]. The similar strategy was used upon the siRNA-mediated knockdown of the beta-site APP cleaving enzyme β-secretase 1 (*BACE1*), a therapeutic target in Alzheimer’s disease. Using dendritic cell-derived EVs tagged with Lamp2b fused to the neuron-targeting peptide rabies virus glycoprotein (RVG), engineered EVs not only crossed the blood–brain barrier but delivered *BACE1*-specific siRNA cargo specifically to neurons, microglia, and oligodendrocytes without losing the functionality of the payload [40]. RGV tagging of EVs seems to be a universally useful approach for biopharmaceutical delivery in neurodegenerative disorders, since these EVs, when loaded with an anti-α-synuclein shRNA construct, were also successfully used to repress α-synuclein expression in Parkinson’s models [146]. Given their utility in neurodegenerative disease, RVG-engineered EVs have also been tested for infectious conditions affecting the CNS [147]. Carrying Zika virus genome-specific siRNA, RVG-engineered EVs successfully crossed both the placental and blood–brain barriers after systemic administration in AG6 mice, an immunocompromised model highly susceptible to Zika virus infection. In the fetal brain, the siRNA cargo suppressed Zika virus replication, reduced neuroinflammation, and prevented virus-induced neurological damage of the developing fetus [147].

Besides small interfering RNA species, it has also been demonstrated that EVs are able to deliver functional mRNAs to force protein synthesis in target cells. Indeed, in a familial hypercholesterolemia model, it has been shown that EV-mediated delivery of the low-density lipoprotein receptor (LDLR)-encoding mRNA was able to restore expression of functional LDLR in hepatocytes of mice lacking LDLR, bringing classical gene therapy closer to clinical practice [148]. EVs have also been tested to deliver functional mRNAs as antimicrobial biopharmaceuticals, as it has been reported in the context of HIV-1 infections. Engineered exosomes loaded with an mRNA construct encoding a zinc finger protein fused to DNA methyltransferase 3A have been explored to generate a fusion protein that targets and methylates the HIV-1 promoter, epigenetically repressing viral gene expression. In humanized mouse models, systemic administration of these exosomes resulted in significant and sustained repression of HIV-1 replication, demonstrating that the concept of the use of EVs as delivery vehicles of new generation antimicrobial biopharmaceuticals is viable [149].

Delivering even more complex nuclei acids, like expression vectors, also seems to be possible via EVs. Indeed, EVs carrying CRISPR/Cas9-encoding plasmids targeting the poly (ADP-ribose) polymerase-1 (*PARP-1*) gene were shown to block PARP-1 in ovarian cancer cells, demonstrating preserved transcriptional activity of the recombinant DNA cargo during delivery [150]. The flexibility of EV-based delivery systems is well demonstrated by data that EVs loaded with more bulky cargo can also deliver the therapeutic payload successfully. Using RVG-engineered exosomes targeting the α7-nicotinic acetyl-choline receptor (α7NAChR) on the surface of the β amyloid peptide-producing N2a neurons loaded with CD10, a variant of neprilysin that degrades amyloid-beta (Aβ) peptides and, thus, is believed to be involved in AD pathology, significant reduction of the secreted Aβ40 levels was achieved in vitro [151]. Moreover, in vivo, the same EV construct accumulated in the hippocampus, accompanied by the repression of proinflammatory genes *IL1A, TNFA,* and *NFKB1*, and induction of the anti-inflammatory gene *IL10*, demonstrating the feasibility of the concept of using EVs to deliver complex biopharmaceuticals directly to cells involved in the pathogenesis of human disorders [151].

The successful delivery of large cargo apparently depends on neither the cell type of origin of EVs nor the mode of administration. Using macrophage-derived EVs, for instance, complete functional enzymes, like the antioxidant catalase, have been shown to be transported into the brain tissue in Parkinson’s disease models, even upon intranasal administration [127]. There, EV-mediated delivery of catalase preserved dopaminergic neurons in the substantia nigra of treated mice, at least in part, via the accompanying reduction of neuroinflammation and oxidative stress. Administration of native catalase, in contrast, had no effect since the unwrapped enzyme was not able to cross the blood–brain barrier and, thus, was rapidly cleared from the circulation [127]. Moreover, autologous dendritic cell (DC)-derived exosomes have also been reported to efficiently transport proteinaceous cargo, like tumor antigens, to cancer tissues, showing that the concept of EV-mediated biopharmaceuticals delivery might fit a wide range of disease scenarios [152].

In accordance, engineered EVs can also be applied in degenerative disorders of complex tissues like bone and muscle. EVs displaying the fusion variant of the MSC-binding peptide E7 and Lamp2b on their surface were shown to selectively target MSCs in exotic environments like the cartilage [153]. This EV-mediated targeted delivery of the chondrocyte differentiation inducer Kartogenin (KGN) allowed for homogenous cytosolic dissemination of the payload, resulting in increased chondrogenesis both in vitro and in vivo [153]. Intra-articular co-administration of synovial fluid MSCs with E7-KGN EVs markedly increased the mRNA and protein levels of chondrogenic markers *SOX9*, *COL2A1*, and *ACAN* in a rat model of osteoarthritis [153]. These experimental data fueled several clinical trials to explore the use of EVs as delivery systems of biopharmaceuticals in various human disorders.

**Table 2:** Summary of EV therapeutics across various disease models, detailing EV sources, cargo types, surface modifications, and observed therapeutic effects.

EV Source	Cargo	Disease Application	EV Surface Modifications	Therapeutic Effect	Reference
Mesenchymal Stem Cell (MSC)-derived exosomes	miR-26a	Hepatocellular Carcinoma	Lamp2b fused with anti-GPC3 single-chain variable fragment (scFv) for targeted delivery to GPC3-positive HCC cells	Downregulation of Cyclin D2 and Cyclin E2 expression, leading to inhibited tumor cell proliferation and suppressed tumor growth in vivo	[145]
Cancer-derived exosomes	CRISPR/Cas9 plasmid targeting *PARP-1*	Ovarian Cancer	None; utilized inherent tumor tropism of cancer-derived exosome	Suppression of PARP-1 expression, leading to apoptosis in ovarian cancer cells and enhanced sensitivity to cisplatin chemotherapy	[150]
Dendritic cell-derived exosomes	siRNA targeting *BACE1*	Alzheimer’s Disease	Lamp2b fused with rabies virus glycoprotein (RVG) peptide for neuron-specific targeting	Achieved 60% mRNA and 62% protein knockdown of BACE1 in the mouse brain, demonstrating effective gene silencing in neurons, microglia, and oligodendrocytes following systemic administration	[40]
Genetically engineered exosomes	Neprilysin variant	Alzheimer’s Disease	Display of RVG peptide for targeting α7 nicotinic acetylcholine receptors (α7-nAChR)	Enhanced degradation of amyloid-beta (Aβ) peptides, reduction of pro-inflammatory cytokines (IL-1α, TNF-α, NF-κB), and increased anti-inflammatory cytokine (IL-10) expression in the hippocampus	[151]
Macrophage-derived exosomes	Catalase enzyme	Parkinson’s Disease	None; utilized natural exosome properties for delivery	Intranasal administration of catalase-loaded exosomes (exoCAT) in a Parkinson’s disease mouse model led to significant neuroprotective effects, including reduced oxidative stress and inflammation, and improved neuronal survival	[127]
HEK293T-derived exosomes	Plasmid DNA encoding shRNA targeting α-synuclein	Parkinson’s Disease	Lamp2b fused with RVG peptide for targeting neurons via nicotinic acetylcholine receptors	Significant reduction of α-synuclein mRNA and protein levels in the enteric nervous system and spinal cord following intravenous administration	[146]
MSC-derived exosomes	mRNA encoding ZFP362-DNMT3A fusion protein (ZPAMt)	Human Immunodeficiency Virus Type 1 Infection	None; utilized natural tropism of MSC-derived exosomes	Induced stable epigenetic repression of HIV-1 by promoting DNA methylation of the viral promoter, leading to sustained suppression of viral replication in humanized mouse models and increased CD4⁺ T-cell counts	[149]
Small EVs from HEK293T cells	Antiviral siRNA targeting Zika virus (ZIKV)	Zika Virus Infection and Microcephaly	Surface display of RVG peptide for targeting neurons via nicotinic acetylcholine receptors	Selective delivery of siRNA to fetal brain, resulting in inhibition of ZIKV infection and mitigation of ZIKV-induced microcephaly in a mouse model	[147]
HEK293T cell-derived EVs	mRNA encoding low-density lipoprotein receptor (LDLR)	Familial Hypercholesterolemia (FH)	Surface functionalization with an ApoB100-derived peptide for targeted delivery to hepatocytes	Restoration of LDLR expression in hepatocytes, leading to enhanced clearance of low-density lipoprotein cholesterol (LDL-C) and amelioration of hypercholesterolemia in FH mouse models	[148]
Engineered exosomes displaying E7 peptide	Kartogenin (KGN)	Degenerative Joint Disease	Fusion of MSC-targeting E7 peptide with exosomal membrane protein Lamp2b	Enhanced chondrogenic differentiation of synovial fluid-derived mesenchymal stem cells (SF-MSCs) and improved cartilage regeneration in vivo	[153]

## 5. Clinical Trials of EVs as Delivery Vehicles of Biopharmaceuticals

The first clinical trial utilizing EVs as delivery vehicles of biopharmaceuticals was conducted in 2005, with a focus on treating metastatic melanoma [154]. This pioneering phase I study aimed to evaluate the safety and feasibility of using autologous dendritic cell-derived exosomes loaded with tumor antigens to stimulate an immune response against melanoma cells, highlighting the potential of EVs as immune-modulating drug delivery systems [154]. Since then, oncology represents a key area for EV-based clinical trials, where these vesicles are being leveraged for both immune modulation and direct tumor targeting. For the former one, EVs are being harnessed as a platform to enhance immunotherapy by delivering tumor-associated antigens to the immune system. This has been tested in the phase 2 trial NCT01159288, in which dendritic cell-derived exosomes loaded with multiple tumor-associated antigens (MAGE-3, MAGE-1, NY-ESO-1, MART-1) were expected to provoke a cytotoxic T-cell response against unresectable non-small cell lung cancer (NSCLC) [155,156]. It was demonstrated that in patients with NSCLC, DC-derived EVs enhanced the NK cell functions while avoiding any detectable induction of antigen-specific T-cell responses [157].

Further expanding this concept, researchers are advancing EV-based immunotherapy by developing chimeric exosomes capable of personalized tumor targeting. In the clinical trial NCT04592484, an indirect immune oncology strategy has been evaluated using HEK-293 cell-derived EVs carrying the stimulator of interferon genes (STING) agonist CDK-002 [158]. STING mediates a key pathway in innate immune sensing, and its activation via EV-based delivery of CDK-002 is expected to boost anti-tumor immunity.

In addition to supporting immune oncology, EVs are also being tested for targeting oncogenesis directly. NCT03608631 is a trial using mesenchymal stromal cell-derived EVs loaded with KrasG12D-specific siRNA in metastatic pancreatic ductal adenocarcinoma patients. The KrasG12D mutation is a major oncogenic driver in pancreatic cancer, and the EVs used in this study are expected to inhibit tumor proliferation and improve patient outcomes via the downregulation of KrasG12D expression in target cells [159].

For directly targeting key components of the oncogenesis, strategies have been tested against other solid cancers like glioblastoma in the NCT01550523 trial, where autologous tumor cell-derived EVs carrying antisense oligodeoxynucleotides targeting IGF-1R, overexpressed in most glioblastomas, were evaluated, expecting significant repression of IGF-1R translation and consequent tumor growth arrest [160,161]. The therapy was not only well tolerated, but it also demonstrated a nearly three-fold increase in the median progression-free survival in patients with glioblastoma multiforme compared to the standard of care [162].

Besides their delivery vehicle role to transport therapeutic agents designed against the target pathology, EVs are being examined for their potential role in mitigating the side effects of canonical treatments like radiotherapy. Testing this concept, NCT01668849 evaluated the protective potential of grape-derived exosomes against chemoradiation-induced oral mucositis in cancer patients of the dental and maxillofacial field. By delivering bioactive molecules such as catechin, which reduce oxidative stress and inflammation by inhibiting TNFα-induced NF-κB signaling, these exosomes may be a non-invasive strategy to mitigate mucosal damage caused by canonical cancer treatments [163,164,165].

Beyond oncology, EVs are being explored for their role in modulating the immune response in inflammatory diseases. NCT04902183 is a phase 2 trial that looked at HEK-293-derived EVs overexpressing CD24 in moderate-to-severe COVID-19 patients. CD24 is known to play a role in immune regulation and inflammation, and the trial demonstrated that CD24 overexpressing EVs were well tolerated, with no treatment-related adverse events; reduced key inflammatory markers by 50% in the majority of patients; and improved the respiratory rate as well as oxygen saturation in COVID-19 patients [166]. A similar concept has been studied in another phase 1 trial, NCT01294072, in which plant-derived exosomes are explored for delivery of the anti-inflammatory curcumin to the intestinal epithelium. As curcumin has poor bioavailability, exosome carriers are expected to improve its stability, absorption, and anti-inflammatory effects in colorectal disorders, exploiting the improved bioavailability of the EV-encapsuled cargo [167].

In addition to their role in immune modulation, EVs are also being explored as delivery tools for tissue repair. NCT05078385 was a phase 1/2a trial of allogeneic MSC-derived EVs containing type VII collagen-encoding mRNA for the use as a topical therapy (AGLE-102) in severe second degree-burned patients. These regenerative EVs were expected to speed up wound healing by stimulating fibroblast activation, angiogenesis, and extracellular matrix remodeling [168]. Within 48 h of a burn injury, a single topical application of AGLE-102 resulted in a significant improvement in scar appearance over a 12-week period, decreased swelling, accelerated wound healing, and no indications of additional tissue damage while no safety issues were noted [169].

A similar regenerative approach was applied to chronic wound healing, where impaired vascularization leads to prolonged tissue damage. NCT04134676 investigated Wharton’s Jelly mesenchymal stem cell-derived EVs, which are naturally enriched with proangiogenic and wound-healing factors (TGF-β, VEGF, IGF-1, IL-6, IL-8), for chronic ulcer wounds. By stimulating angiogenesis and epithelialization, these EVs were anticipated to enhance tissue repair and accelerate wound closure in patients with chronic non-healing ulcers. It was demonstrated that topical administration of 10% secretome from human umbilical cord mesenchymal stem cells (SC-hUCMSCs) effectively supported wound healing, particularly in chronic ulcers resulting from leprosy and diabetes. Following treatment, there were noticeable decreases in the length, width, and overall area of the wound [170].

Another intriguing area of application is the field of inherited metabolic disorders, where EVs can act as delivery vehicles for genetic material. One such application of EV-based gene therapy is the treatment of homozygous familial hypercholesterolemia (HFH), a severe genetic disorder where the low-density lipoprotein receptor (*LDLR*) gene is either deleted or showing a loss-of-function mutation. The consequently compromised cellular uptake of LDL cholesterol results in high LDL levels in the blood and, thus, premature cardiovascular disease. NCT05043181, a phase 1 trial using bone marrow mesenchymal stromal cell-derived EVs loaded with *LDLR* mRNA, evaluates if LDL receptor expression can be restored in hepatocytes [145]. Table 3 presents a summary of the clinical trials that have or currently are investigating the role of EVs as biopharmaceutical delivery vehicles. These studies highlight the potential of EVs to encapsulate and protect therapeutic agents, enhancing their stability and targeted delivery in vivo. 

## 6. Challenges and Future Perspectives

Translating EVs into the clinicals aspects of medicine requires solving several challenges. One of the biggest hurdles in their clinical translation is the lack of standardized, scalable production methods that meet regulatory requirements. Current isolation methods like ultracentrifugation supply low yields and can damage EVs or introduce contaminants [79]. Variability in laboratory protocols makes it hard to maintain consistent EV properties. For scaling purposes, bioreactor-based cell cultures offer promise, but optimizing culture conditions remains a challenge [176]. Features like cell density, nutrient depletion, hypoxia, and stress induction can increase EV yield but may also compromise bioactivity and homogeneity [177]. Novel, more efficient and scalable methods like tangential flow filtration may present a solution providing higher yields and reduced processing time compared to ultracentrifugation [178]. Besides the ongoing innovation to improve EV yields upon their biogenesis, quantification of EVs is another key aspect when it comes to determining therapeutic doses, and better analytical methods are needed to measure EVs accurately and reproducibly.

Functionalizing EVs for targeted delivery is one of the biggest advantages of their use for precision medicine, although it also raises issues of off-target delivery and reduced efficacy [179]. Recent research investigating the use of hydrogels and other sustained-release platforms to boost the effectiveness of EV-based therapies, providing prolonged and controlled release of their payload, may address reduced efficacy issues. Indeed, EVs in hydrogel matrices deliver more cargo, and their release is sustained longer than bolus doses, an appealing characteristic for chronic disease management and tissue regeneration applications [180,181].

Maintaining EV stability during storage and transport also poses a challenge. Conventional methods, e.g., freezing, are expensive and impact physical and biological characteristics of EVs. Innovations such as adding alginate to prevent cryoinjury are progressing with advancements in research, but simpler and more cost-effective storage solutions are required to make EVs more clinically relevant for the future [182].

Although there is much promise with clinical therapeutic work involving EVs, they ultimately are not “safe” per se. Tumor-derived EVs, for example, pose a risk of tumorigenesis, metastasis, and angiogenesis [183]. Immunogenicity, immunotoxicity, and carcinogenicity must be understood and mitigated before EVs can be used in the clinic [183,184]. These limitations of natural EVs gave birth to the need for developing certain transformative strategies, like hybrid vehicles, biomimetic, and nanoparticle integration approaches. All techniques effectively combined the biological advantages offered by EVs with the flexibility and scalability of synthetic platforms, leading to new strategic approaches for drug delivery. Hybrid vesicles, for instance, combine EVs with liposomes/nanoparticles that enhance stability, flexibility, and targeting. Indeed, EV–liposome hybrids loaded with chemotherapeutics presented enhanced therapeutic efficacy and improved drug release profiles [185]. In contrast, biomimetic vesicles are engineered vehicles that retain EV-like structures while allowing for the integration of advanced targeting mechanisms, scalable production, and precise design [186]. Engineered leukocyte-mimicking nanovesicles, “leukosomes”, for instance, can deliver doxorubicin for breast cancer and melanoma tumor treatment like that of their EV counterparts [187].

## 7. Conclusions

Biopharmaceuticals have been currently transforming modern medicine. They have given us specific, patient-focused treatments for complex diseases. From monoclonal antibodies and mRNA vaccines to oligonucleotides and protein-based treatments, these innovations are now addressing the gaps in precision medicine. Courtesy of the continuous innovation and collaboration across various scientific disciplines, EVs as drug delivery vehicles for biopharmaceuticals are on course to revolutionize the management of multifaceted medical challenges into highly personalized and efficient healthcare solutions.

## Figures and Tables

**Table 3:** Summary of completed * and ongoing clinical trials investigating extracellular vesicle-based delivery of biopharmaceuticals.

Body System	Clinical Trial ID and Phase	EV Source	EV Cargo	Purpose	Reference
Cardiovascular (Homozygous Familial Hypercholesterolemia—HoFH)	NCT05043181—Phase 1 (*n* = 30)	Bone Marrow Mesenchymal Stromal Cell-EVs	Low-density lipoprotein (LDL) mRNA	Safety and effectiveness of exosome-mRNA therapy in HoFH	[171]
Central Nervous System (Recurrent Glioblastoma)	NCT01550523 *—Phase 1 (*n* = 13)	Autologous tumor cells	Antisense oligodeoxynucleotides (IGF-1R AS ODN)	Stimulate immune response in glioblastoma	[160,172]
Gastrointestinal (Colon)	NCT01294072—Phase 1 (*n* = 35)	Plant-derived exosomes	Curcumin	Plant exosomes for curcumin delivery to colon tissue	[167]
Gastrointestinal (Metastatic Pancreatic Ductal Adenocarcinoma)	NCT03608631—Interventional (*n* = 15)	Mesenchymal stromal cells	KrasG12D siRNA	MSC exosomes for Kras mutation in pancreatic cancer	[159]
Immunological (Unresectable Non-Small Cell Lung Cancer)	NCT01159288 *—Phase 2 (*n* = 41)	Dendritic cell-derived exosomes	Tumor antigens (MAGE-3 DP04, MAGE-1 A2, MAGE-3 A2, NY-ESO-1, MART-1 A2)	Assess exosome vaccines in NSCLC	[173]
Integumentary (Skin; Severe Second-Degree Burns)	NCT05078385 *—Phase 1/2a (*n* = 1)	Allogeneic Mesenchymal Stem Cells	AGLE-102 (COL7A1 mRNA)	MSC exosomes for severe burns	[174]
Oral/Dental (Prevention of Chemoradiation-associated Oral Mucositis)	NCT01668849 *—Phase 1 (*n* = 60)	Edible plant-derived exosomes	Grape exosomes	Effects of plant exosomes on chemoradiation-associated oral mucositis	[163]
Respiratory (COVID-19)	NCT04902183—Phase 2 (*n* = 90)	Human Embryonic Kidney (HEK)-293 cells	CD24 overexpressed exosomes	CD24 exosomes for inflammation in COVID-19	[166]
Skin (Chronic Ulcer Wounds)	NCT04134676 *—Phase 1 (*n* = 38)	Wharton’s Jelly MSCs	Proangiogenic and wound healing promoting factors (TGF-B, VEGF, IGF-1, IL-6, IL-8)	Conditioned medium for chronic wound healing	[175]
Various Advanced/Metastatic, Recurrent, Injectable Solid Tumors	NCT04592484 *—Phase 1/2 (*n* = 27)	HEK-293 cells	Intratumoral injection of CDK-002 (STING agonist)	Intratumoral injection for solid tumors	[158]

## Data Availability

No new datasets were generated for this review. All referenced studies are cited in the manuscript.

## References

[1] Amir E., Miller N., Geddie W., Freedman O., Kassam F., Simmons C., Oldfield M., Dranitsaris G., Tomlinson G., Laupacis A. (2012). Prospective study evaluating the impact of tissue confirmation of metastatic disease in patients with breast cancer. J. Clin. Oncol..

[2] Chen S., Li S., Zhang J., Zhang L., Chen Y., Wang L., Jin L., Hu Y., Qi X., Huang H. (2018). Preimplantation Genetic Diagnosis of Multiple Endocrine Neoplasia Type 2A Using Informative Markers Identified by Targeted Sequencing. Thyroid.

[3] Fu X., Ying J., Yang L., Fang W., Han W., Hu H., Zhang S., Yuan Y. (2023). Dual targeted therapy with pyrotinib and trastuzumab for HER2-positive advanced colorectal cancer: A phase 2 trial. Cancer Sci..

[4] U.S. Food & Drug Administration What Are “Biologics” Questions and Answers. https://www.fda.gov/about-fda/center-biologics-evaluation-and-research-cber/what-are-biologics-questions-and-answers.

[5] National Center for Biotechnology Information PubChem Compound Summary for CID 70678557, Insulin. https://pubchem.ncbi.nlm.nih.gov/compound/Insulin.

[6] National Center for Biotechnology Information PubChem Compound Summary for CID 2244, Aspirin. https://pubchem.ncbi.nlm.nih.gov/compound/Aspirin.

[7] Saffran M., Kumar G.S., Savariar C., Burnham J.C., Williams F., Neckers D.C. (1986). A new approach to the oral administration of insulin and other peptide drugs. Science.

[8] Schilling R.J., Mitra A.K. (1991). Degradation of insulin by trypsin and alpha-chymotrypsin. Pharm. Res..

[9] Boyes R.N., Scott D.B., Jebson P.J., Godman M.J., Julian D.G. (1971). Pharmacokinetics of lidocaine in man. Clin. Pharmacol. Ther..

[10] Fincke A., Winter J., Bunte T., Olbrich C. (2014). Thermally induced degradation pathways of three different antibody-based drug development candidates. Eur. J. Pharm. Sci..

[11] Back J.F., Oakenfull D., Smith M.B. (1979). Increased thermal stability of proteins in the presence of sugars and polyols. Biochemistry.

[12] Tobio M., Sanchez A., Vila A., Soriano I.I., Evora C., Vila-Jato J.L., Alonso M.J. (2000). The role of PEG on the stability in digestive fluids and in vivo fate of PEG-PLA nanoparticles following oral administration. Colloids Surf. B Biointerfaces.

[13] Csaba N., Sanchez A., Alonso M.J. (2006). PLGA:poloxamer and PLGA:poloxamine blend nanostructures as carriers for nasal gene delivery. J. Control. Release.

[14] de Souza G.A., Godoy L.M., Mann M. (2006). Identification of 491 proteins in the tear fluid proteome reveals a large number of proteases and protease inhibitors. Genome Biol..

[15] Lee V.H., Chien D.S., Sasaki H. (1988). Ocular ketone reductase distribution and its role in the metabolism of ocularly applied levobunolol in the pigmented rabbit. J. Pharmacol. Exp. Ther..

[16] Guo Y., Zhang Y., Ma J., Li Q., Li Y., Zhou X., Zhao D., Song H., Chen Q., Zhu X. (2018). Light/magnetic hyperthermia triggered drug released from multi-functional thermo-sensitive magnetoliposomes for precise cancer synergetic theranostics. J. Control. Release.

[17] Vroman L. (1962). Effect of absorbed proteins on the wettability of hydrophilic and hydrophobic solids. Nature.

[18] Gu Y., Xu C., Wang Y., Zhou X., Fang L., Cao F. (2019). Multifunctional Nanocomposites Based on Liposomes and Layered Double Hydroxides Conjugated with Glycylsarcosine for Efficient Topical Drug Delivery to the Posterior Segment of the Eye. Mol. Pharm..

[19] Chargaff E., West R. (1946). The biological significance of the thromboplastic protein of Wood. J. Biol. Chem..

[20] Admyre C., Johansson S.M., Qazi K.R., Filen J.J., Lahesmaa R., Norman M., Neve E.P., Scheynius A., Gabrielsson S. (2007). Exosomes with immune modulatory features are present in human breast milk. J. Immunol..

[21] Ogawa Y., Kanai-Azuma M., Akimoto Y., Kawakami H., Yanoshita R. (2008). Exosome-like vesicles with dipeptidyl peptidase IV in human saliva. Biol. Pharm. Bull..

[22] Théry C., Witwer K.W., Aikawa E., Alcaraz M.J., Anderson J.D., Andriantsitohaina R., Antoniou A., Arab T., Archer F., Atkin-Smith G.K. (2018). Minimal information for studies of extracellular vesicles 2018 (MISEV2018): A position statement of the International Society for Extracellular Vesicles and update of the MISEV2014 guidelines. J. Extracell. Vesicles.

[23] Harding C., Heuser J., Stahl P. (1983). Receptor-mediated endocytosis of transferrin and recycling of the transferrin receptor in rat reticulocytes. J. Cell Biol..

[24] Pan B.T., Teng K., Wu C., Adam M., Johnstone R.M. (1985). Electron microscopic evidence for externalization of the transferrin receptor in vesicular form in sheep reticulocytes. J. Cell Biol..

[25] Katzmann D.J., Babst M., Emr S.D. (2001). Ubiquitin-dependent sorting into the multivesicular body pathway requires the function of a conserved endosomal protein sorting complex, ESCRT-I. Cell.

[26] Raymond C.K., Howald-Stevenson I., Vater C.A., Stevens T.H. (1992). Morphological classification of the yeast vacuolar protein sorting mutants: Evidence for a prevacuolar compartment in class E vps mutants. Mol. Biol. Cell.

[27] Babst M., Sato T.K., Banta L.M., Emr S.D. (1997). Endosomal transport function in yeast requires a novel AAA-type ATPase, Vps4p. EMBO J..

[28] Escola J.M., Kleijmeer M.J., Stoorvogel W., Griffith J.M., Yoshie O., Geuze H.J. (1998). Selective enrichment of tetraspan proteins on the internal vesicles of multivesicular endosomes and on exosomes secreted by human B-lymphocytes. J. Biol. Chem..

[29] Thery C., Regnault A., Garin J., Wolfers J., Zitvogel L., Ricciardi-Castagnoli P., Raposo G., Amigorena S. (1999). Molecular characterization of dendritic cell-derived exosomes. Selective accumulation of the heat shock protein hsc73. J. Cell Biol..

[30] Valadi H., Ekstrom K., Bossios A., Sjostrand M., Lee J.J., Lotvall J.O. (2007). Exosome-mediated transfer of mRNAs and microRNAs is a novel mechanism of genetic exchange between cells. Nat. Cell Biol..

[31] Lancaster G.I., Febbraio M.A. (2005). Exosome-dependent trafficking of HSP70: A novel secretory pathway for cellular stress proteins. J. Biol. Chem..

[32] Nabhan J.F., Hu R., Oh R.S., Cohen S.N., Lu Q. (2012). Formation and release of arrestin domain-containing protein 1-mediated microvesicles (ARMMs) at plasma membrane by recruitment of TSG101 protein. Proc. Natl. Acad. Sci. USA.

[33] Taylor J., Azimi I., Monteith G., Bebawy M. (2020). Ca^2+^ mediates extracellular vesicle biogenesis through alternate pathways in malignancy. J. Extracell. Vesicles.

[34] Muralidharan-Chari V., Clancy J., Plou C., Romao M., Chavrier P., Raposo G., D’Souza-Schorey C. (2009). ARF6-regulated shedding of tumor cell-derived plasma membrane microvesicles. Curr. Biol..

[35] Lo Cicero A., Majkowska I., Nagase H., Di Liegro I., Troeberg L. (2012). Microvesicles shed by oligodendroglioma cells and rheumatoid synovial fibroblasts contain aggrecanase activity. Matrix Biol..

[36] Wei X., Liu C., Wang H., Wang L., Xiao F., Guo Z., Zhang H. (2016). Surface Phosphatidylserine Is Responsible for the Internalization on Microvesicles Derived from Hypoxia-Induced Human Bone Marrow Mesenchymal Stem Cells into Human Endothelial Cells. PLoS ONE.

[37] Kanada M., Bachmann M.H., Hardy J.W., Frimannson D.O., Bronsart L., Wang A., Sylvester M.D., Schmidt T.L., Kaspar R.L., Butte M.J. (2015). Differential fates of biomolecules delivered to target cells via extracellular vesicles. Proc. Natl. Acad. Sci. USA.

[38] Tian T., Zhu Y.L., Hu F.H., Wang Y.Y., Huang N.P., Xiao Z.D. (2013). Dynamics of exosome internalization and trafficking. J. Cell Physiol..

[39] Somiya M., Kuroda S.i. (2022). Verification of extracellular vesicle-mediated functional mRNA delivery via RNA editing. bioRxiv.

[40] Alvarez-Erviti L., Seow Y., Yin H., Betts C., Lakhal S., Wood M.J. (2011). Delivery of siRNA to the mouse brain by systemic injection of targeted exosomes. Nat. Biotechnol..

[41] Huang W., Qu M., Li L., Liu T., Lin M., Yu X. (2021). SiRNA in MSC-derived exosomes silences CTGF gene for locomotor recovery in spinal cord injury rats. Stem Cell Res. Ther..

[42] Kim Y.K., Hong Y., Bae Y.R., Goo J., Kim S.A., Choi Y., Nam G.H., Kwon M., Yun S.G., Lee G. (2022). Advantage of extracellular vesicles in hindering the CD47 signal for cancer immunotherapy. J. Control. Release.

[43] Kamerkar S., LeBleu V.S., Sugimoto H., Yang S., Ruivo C.F., Melo S.A., Lee J.J., Kalluri R. (2017). Exosomes facilitate therapeutic targeting of oncogenic KRAS in pancreatic cancer. Nature.

[44] Kooijmans S.A.A., Fliervoet L.A.L., van der Meel R., Fens M., Heijnen H.F.G., van Bergen En Henegouwen P.M.P., Vader P., Schiffelers R.M. (2016). PEGylated and targeted extracellular vesicles display enhanced cell specificity and circulation time. J. Control. Release.

[45] Lai R.C., Arslan F., Lee M.M., Sze N.S., Choo A., Chen T.S., Salto-Tellez M., Timmers L., Lee C.N., El Oakley R.M. (2010). Exosome secreted by MSC reduces myocardial ischemia/reperfusion injury. Stem Cell Res..

[46] Zhang L., Graf I., Kuang Y., Zheng X., Haupt M., Majid A., Kilic E., Hermann D.M., Psychogios M.N., Weber M.S. (2021). Neural Progenitor Cell-Derived Extracellular Vesicles Enhance Blood-Brain Barrier Integrity by NF-κB (Nuclear Factor-kappaB)-Dependent Regulation of ABCB1 (ATP-Binding Cassette Transporter B1) in Stroke Mice. Arterioscler. Thromb. Vasc. Biol..

[47] Tan J., Wu W., Xu X., Liao L., Zheng F., Messinger S., Sun X., Chen J., Yang S., Cai J. (2012). Induction therapy with autologous mesenchymal stem cells in living-related kidney transplants: A randomized controlled trial. JAMA.

[48] Haraszti R.A., Miller R., Stoppato M., Sere Y.Y., Coles A., Didiot M.C., Wollacott R., Sapp E., Dubuke M.L., Li X. (2018). Exosomes Produced from 3D Cultures of MSCs by Tangential Flow Filtration Show Higher Yield and Improved Activity. Mol. Ther..

[49] Harrell C.R., Jovicic N., Djonov V., Arsenijevic N., Volarevic V. (2019). Mesenchymal Stem Cell-Derived Exosomes and Other Extracellular Vesicles as New Remedies in the Therapy of Inflammatory Diseases. Cells.

[50] Grange C., Tapparo M., Bruno S., Chatterjee D., Quesenberry P.J., Tetta C., Camussi G. (2014). Biodistribution of mesenchymal stem cell-derived extracellular vesicles in a model of acute kidney injury monitored by optical imaging. Int. J. Mol. Med..

[51] Katakowski M., Buller B., Zheng X., Lu Y., Rogers T., Osobamiro O., Shu W., Jiang F., Chopp M. (2013). Exosomes from marrow stromal cells expressing miR-146b inhibit glioma growth. Cancer Lett..

[52] Gennai S., Monsel A., Hao Q., Park J., Matthay M.A., Lee J.W. (2015). Microvesicles Derived From Human Mesenchymal Stem Cells Restore Alveolar Fluid Clearance in Human Lungs Rejected for Transplantation. Am. J. Transplant..

[53] Bruno S., Grange C., Deregibus M.C., Calogero R.A., Saviozzi S., Collino F., Morando L., Busca A., Falda M., Bussolati B. (2009). Mesenchymal stem cell-derived microvesicles protect against acute tubular injury. J. Am. Soc. Nephrol..

[54] Tieu A., Stewart D.J., Chwastek D., Lansdell C., Burger D., Lalu M.M. (2023). Biodistribution of mesenchymal stromal cell-derived extracellular vesicles administered during acute lung injury. Stem Cell Res. Ther..

[55] Zhong L., Wang J., Wang P., Liu X., Liu P., Cheng X., Cao L., Wu H., Chen J., Zhou L. (2023). Neural stem cell-derived exosomes and regeneration: Cell-free therapeutic strategies for traumatic brain injury. Stem Cell Res. Ther..

[56] Qian C., Wang Y., Ji Y., Chen D., Wang C., Zhang G., Wang Y. (2022). Neural stem cell-derived exosomes transfer miR-124-3p into cells to inhibit glioma growth by targeting FLOT2. Int. J. Oncol..

[57] Lagos-Quintana M., Rauhut R., Yalcin A., Meyer J., Lendeckel W., Tuschl T. (2002). Identification of tissue-specific microRNAs from mouse. Curr. Biol..

[58] Joshi B.S., Zuhorn I.S. (2021). Heparan sulfate proteoglycan-mediated dynamin-dependent transport of neural stem cell exosomes in an in vitro blood-brain barrier model. Eur. J. Neurosci..

[59] Graham F.L., Smiley J., Russell W.C., Nairn R. (1977). Characteristics of a human cell line transformed by DNA from human adenovirus type 5. J. Gen. Virol..

[60] Lin Y.C., Boone M., Meuris L., Lemmens I., Van Roy N., Soete A., Reumers J., Moisse M., Plaisance S., Drmanac R. (2014). Genome dynamics of the human embryonic kidney 293 lineage in response to cell biology manipulations. Nat. Commun..

[61] Li J., Chen X., Yi J., Liu Y., Li D., Wang J., Hou D., Jiang X., Zhang J., Wang J. (2016). Identification and Characterization of 293T Cell-Derived Exosomes by Profiling the Protein, mRNA and MicroRNA Components. PLoS ONE.

[62] Dellgren C., Nehlin J.O., Barington T. (2015). Cell surface expression level variation between two common Human Leukocyte Antigen alleles, HLA-A2 and HLA-B8, is dependent on the structure of the C terminal part of the alpha 2 and the alpha 3 domains. PLoS ONE.

[63] Giles J.R., Globig A.M., Kaech S.M., Wherry E.J. (2023). CD8^+^ T cells in the cancer-immunity cycle. Immunity.

[64] Betker J.L., Angle B.M., Graner M.W., Anchordoquy T.J. (2019). The Potential of Exosomes from Cow Milk for Oral Delivery. J. Pharm. Sci..

[65] Samuel M., Chisanga D., Liem M., Keerthikumar S., Anand S., Ang C.S., Adda C.G., Versteegen E., Jois M., Mathivanan S. (2017). Bovine milk-derived exosomes from colostrum are enriched with proteins implicated in immune response and growth. Sci. Rep..

[66] Quan S., Du C., Wang K., Nan X., Xiong B. (2022). Different Diets Change Milk Extracellular Vesicle-Protein Profile in Lactating Cows. Agriculture.

[67] Castellani S., Basirico L., Maggiolino A., Lecchi C., De Palo P., Bernabucci U. (2025). Effects of milk extracellular vesicles from Holstein Friesian and Brown Swiss heat-stressed dairy cows on bovine mammary epithelial cells. J. Dairy. Sci..

[68] Xiao Q., Zhao W., Wu C., Wang X., Chen J., Shi X., Sha S., Li J., Liang X., Yang Y. (2022). Lemon-Derived Extracellular Vesicles Nanodrugs Enable to Efficiently Overcome Cancer Multidrug Resistance by Endocytosis-Triggered Energy Dissipation and Energy Production Reduction. Adv. Sci..

[69] Luo L., Wu Z., Wang Y., Li H. (2021). Regulating the production and biological function of small extracellular vesicles: Current strategies, applications and prospects. J. Nanobiotechnol..

[70] Kojima R., Bojar D., Rizzi G., Hamri G.C., El-Baba M.D., Saxena P., Auslander S., Tan K.R., Fussenegger M. (2018). Designer exosomes produced by implanted cells intracerebrally deliver therapeutic cargo for Parkinson’s disease treatment. Nat. Commun..

[71] Xiong Y., Tang R., Xu J., Jiang W., Gong Z., Zhang L., Li X., Ning Y., Huang P., Xu J. (2022). Sequential transplantation of exosomes and mesenchymal stem cells pretreated with a combination of hypoxia and Tongxinluo efficiently facilitates cardiac repair. Stem Cell Res. Ther..

[72] Kumar A., Deep G. (2020). Hypoxia in tumor microenvironment regulates exosome biogenesis: Molecular mechanisms and translational opportunities. Cancer Lett..

[73] Mashouri L., Yousefi H., Aref A.R., Ahadi A.M., Molaei F., Alahari S.K. (2019). Exosomes: Composition, biogenesis, and mechanisms in cancer metastasis and drug resistance. Mol. Cancer.

[74] Gupta S., Rawat S., Krishnakumar V., Rao E.P., Mohanty S. (2022). Hypoxia preconditioning elicit differential response in tissue-specific MSCs via immunomodulation and exosomal secretion. Cell Tissue Res..

[75] Dorayappan K.D.P., Wanner R., Wallbillich J.J., Saini U., Zingarelli R., Suarez A.A., Cohn D.E., Selvendiran K. (2018). Hypoxia-induced exosomes contribute to a more aggressive and chemoresistant ovarian cancer phenotype: A novel mechanism linking STAT3/Rab proteins. Oncogene.

[76] Song Y., Dou H., Li X., Zhao X., Li Y., Liu D., Ji J., Liu F., Ding L., Ni Y. (2017). Exosomal miR-146a Contributes to the Enhanced Therapeutic Efficacy of Interleukin-1beta-Primed Mesenchymal Stem Cells Against Sepsis. Stem Cells.

[77] Kim M., Shin D.I., Choi B.H., Min B.H. (2021). Exosomes from IL-1beta-Primed Mesenchymal Stem Cells Inhibited IL-1beta- and TNF-alpha-Mediated Inflammatory Responses in Osteoarthritic SW982 Cells. Tissue Eng. Regen. Med..

[78] Nakao Y., Fukuda T., Zhang Q., Sanui T., Shinjo T., Kou X., Chen C., Liu D., Watanabe Y., Hayashi C. (2021). Exosomes from TNF-α-treated human gingiva-derived MSCs enhance M2 macrophage polarization and inhibit periodontal bone loss. Acta Biomater..

[79] Coughlan C., Bruce K.D., Burgy O., Boyd T.D., Michel C.R., Garcia-Perez J.E., Adame V., Anton P., Bettcher B.M., Chial H.J. (2020). Exosome Isolation by Ultracentrifugation and Precipitation and Techniques for Downstream Analyses. Curr. Protoc. Cell Biol..

[80] Linares R., Tan S., Gounou C., Arraud N., Brisson A.R. (2015). High-speed centrifugation induces aggregation of extracellular vesicles. J. Extracell. Vesicles.

[81] Livshits M.A., Khomyakova E., Evtushenko E.G., Lazarev V.N., Kulemin N.A., Semina S.E., Generozov E.V., Govorun V.M. (2015). Isolation of exosomes by differential centrifugation: Theoretical analysis of a commonly used protocol. Sci. Rep..

[82] Guo P., Busatto S., Huang J., Morad G., Moses M.A. (2021). A facile magnetic extrusion method for preparing endosome-derived vesicles for cancer drug delivery. Adv. Funct. Mater..

[83] Wen Y., Fu Q., Soliwoda A., Zhang S., Zheng M., Mao W., Wan Y. (2022). Cell-derived nanovesicles prepared by membrane extrusion are good substitutes for natural extracellular vesicles. Extracell. Vesicle.

[84] Shu S., Yang Y., Allen C.L., Hurley E., Tung K.H., Minderman H., Wu Y., Ernstoff M.S. (2020). Purity and yield of melanoma exosomes are dependent on isolation method. J. Extracell. Vesicles.

[85] Gamez-Valero A., Monguio-Tortajada M., Carreras-Planella L., Franquesa M., Beyer K., Borras F.E. (2016). Size-Exclusion Chromatography-based isolation minimally alters Extracellular Vesicles’ characteristics compared to precipitating agents. Sci. Rep..

[86] Welton J.L., Webber J.P., Botos L.A., Jones M., Clayton A. (2015). Ready-made chromatography columns for extracellular vesicle isolation from plasma. J. Extracell. Vesicles.

[87] Corso G., Mager I., Lee Y., Gorgens A., Bultema J., Giebel B., Wood M.J.A., Nordin J.Z., Andaloussi S.E. (2017). Reproducible and scalable purification of extracellular vesicles using combined bind-elute and size exclusion chromatography. Sci. Rep..

[88] Dragovic R.A., Gardiner C., Brooks A.S., Tannetta D.S., Ferguson D.J., Hole P., Carr B., Redman C.W., Harris A.L., Dobson P.J. (2011). Sizing and phenotyping of cellular vesicles using Nanoparticle Tracking Analysis. Nanomedicine.

[89] Wang L., Skotland T., Berge V., Sandvig K., Llorente A. (2017). Exosomal proteins as prostate cancer biomarkers in urine: From mass spectrometry discovery to immunoassay-based validation. Eur. J. Pharm. Sci..

[90] Yokoyama S., Takeuchi A., Yamaguchi S., Mitani Y., Watanabe T., Matsuda K., Hotta T., Shively J.E., Yamaue H. (2017). Clinical implications of carcinoembryonic antigen distribution in serum exosomal fraction-Measurement by ELISA. PLoS ONE.

[91] Skotland T., Sandvig K., Llorente A. (2017). Lipids in exosomes: Current knowledge and the way forward. Prog. Lipid Res..

[92] Cheng L., Sharples R.A., Scicluna B.J., Hill A.F. (2014). Exosomes provide a protective and enriched source of miRNA for biomarker profiling compared to intracellular and cell-free blood. J. Extracell. Vesicles.

[93] Khodakov D., Wang C., Zhang D.Y. (2016). Diagnostics based on nucleic acid sequence variant profiling: PCR, hybridization, and NGS approaches. Adv. Drug Deliv. Rev..

[94] Butler H.J., Ashton L., Bird B., Cinque G., Curtis K., Dorney J., Esmonde-White K., Fullwood N.J., Gardner B., Martin-Hirsch P.L. (2016). Using Raman spectroscopy to characterize biological materials. Nat. Protoc..

[95] Parisse P., Rago I., Ulloa Severino L., Perissinotto F., Ambrosetti E., Paoletti P., Ricci M., Beltrami A.P., Cesselli D., Casalis L. (2017). Atomic force microscopy analysis of extracellular vesicles. Eur. Biophys. J..

[96] Sharma S., Rasool H.I., Palanisamy V., Mathisen C., Schmidt M., Wong D.T., Gimzewski J.K. (2010). Structural-mechanical characterization of nanoparticle exosomes in human saliva, using correlative AFM, FESEM, and force spectroscopy. ACS Nano.

[97] Jung M.K., Mun J.Y. (2018). Sample Preparation and Imaging of Exosomes by Transmission Electron Microscopy. J. Vis. Exp..

[98] Morales-Kastresana A., Jones J.C. (2017). Flow Cytometric Analysis of Extracellular Vesicles. Methods Mol. Biol..

[99] Kanwar S.S., Dunlay C.J., Simeone D.M., Nagrath S. (2014). Microfluidic device (ExoChip) for on-chip isolation, quantification and characterization of circulating exosomes. Lab Chip.

[100] Yang F., Liao X., Tian Y., Li G. (2017). Exosome separation using microfluidic systems: Size-based, immunoaffinity-based and dynamic methodologies. Biotechnol. J..

[101] Makawita S., Diamandis E.P. (2010). The bottleneck in the cancer biomarker pipeline and protein quantification through mass spectrometry-based approaches: Current strategies for candidate verification. Clin. Chem..

[102] Liangsupree T., Multia E., Saarinen J., Ruiz-Jimenez J., Kemell M., Riekkola M.L. (2022). Raman spectroscopy combined with comprehensive gas chromatography for label-free characterization of plasma-derived extracellular vesicle subpopulations. Anal. Biochem..

[103] Wu Y., Deng W., Klinke D.J. (2015). Exosomes: Improved methods to characterize their morphology, RNA content, and surface protein biomarkers. Analyst.

[104] Varga Z., Yuana Y., Grootemaat A.E., van der Pol E., Gollwitzer C., Krumrey M., Nieuwland R. (2014). Towards traceable size determination of extracellular vesicles. J. Extracell. Vesicles.

[105] Cavallaro S., Haag P., Viktorsson K., Krozer A., Fogel K., Lewensohn R., Linnros J., Dev A. (2021). Comparison and optimization of nanoscale extracellular vesicle imaging by scanning electron microscopy for accurate size-based profiling and morphological analysis. Nanoscale Adv..

[106] Han Y., Jones T.W., Dutta S., Zhu Y., Wang X., Narayanan S.P., Fagan S.C., Zhang D. (2021). Overview and Update on Methods for Cargo Loading into Extracellular Vesicles. Processes.

[107] Xu C., Zhai Z., Ying H., Lu L., Zhang J., Zeng Y. (2022). Curcumin primed ADMSCs derived small extracellular vesicle exert enhanced protective effects on osteoarthritis by inhibiting oxidative stress and chondrocyte apoptosis. J. Nanobiotechnol..

[108] Wang X., Li D., Li G., Chen J., Yang Y., Bian L., Zhou J., Wu Y., Chen Y. (2024). Enhanced Therapeutic Potential of Hybrid Exosomes Loaded with Paclitaxel for Cancer Therapy. Int. J. Mol. Sci..

[109] Zhang J., Ji C., Zhang H., Shi H., Mao F., Qian H., Xu W., Wang D., Pan J., Fang X. (2022). Engineered neutrophil-derived exosome-like vesicles for targeted cancer therapy. Sci. Adv..

[110] Benchimol M.J., Hsu M.J., Schutt C.E., Hall D.J., Mattrey R.F., Esener S.C. (2013). Phospholipid/Carbocyanine Dye-Shelled Microbubbles as Ultrasound-Modulated Fluorescent Contrast Agents. Soft Matter.

[111] Schutt C.E., Ibsen S., Benchimol M., Hsu M., Esener S. (2015). Optical detection of harmonic oscillations in fluorescent dye-loaded microbubbles ensonified by ultrasound. Opt. Lett..

[112] Bronisz A., Wang Y., Nowicki M.O., Peruzzi P., Ansari K., Ogawa D., Balaj L., De Rienzo G., Mineo M., Nakano I. (2014). Extracellular vesicles modulate the glioblastoma microenvironment via a tumor suppression signaling network directed by miR-1. Cancer Res..

[113] Li D., Huang S., Zhu J., Hu T., Han Z., Zhang S., Zhao J., Chen F., Lei P. (2019). Exosomes from MiR-21-5p-Increased Neurons Play a Role in Neuroprotection by Suppressing Rab11a-Mediated Neuronal Autophagy In Vitro After Traumatic Brain Injury. Med. Sci. Monit..

[114] Monfared H., Jahangard Y., Nikkhah M., Mirnajafi-Zadeh J., Mowla S.J. (2019). Potential Therapeutic Effects of Exosomes Packed With a miR-21-Sponge Construct in a Rat Model of Glioblastoma. Front. Oncol..

[115] Li X., Liu L.L., Yao J.L., Wang K., Ai H. (2019). Human Umbilical Cord Mesenchymal Stem Cell-Derived Extracellular Vesicles Inhibit Endometrial Cancer Cell Proliferation and Migration through Delivery of Exogenous miR-302a. Stem Cells Int..

[116] Zhang H., Wang Y., Bai M., Wang J., Zhu K., Liu R., Ge S., Li J., Ning T., Deng T. (2018). Exosomes serve as nanoparticles to suppress tumor growth and angiogenesis in gastric cancer by delivering hepatocyte growth factor siRNA. Cancer Sci..

[117] Qiu Y., Sun J., Qiu J., Chen G., Wang X., Mu Y., Li K., Wang W. (2020). Antitumor Activity of Cabazitaxel and MSC-TRAIL Derived Extracellular Vesicles in Drug-Resistant Oral Squamous Cell Carcinoma. Cancer Manag. Res..

[118] Haney M.J., Zhao Y., Jin Y.S., Li S.M., Bago J.R., Klyachko N.L., Kabanov A.V., Batrakova E.V. (2020). Macrophage-Derived Extracellular Vesicles as Drug Delivery Systems for Triple Negative Breast Cancer (TNBC) Therapy. J. Neuroimmune Pharmacol..

[119] Wiklander O.P.B., Mamand D.R., Mohammad D.K., Zheng W., Jawad Wiklander R., Sych T., Zickler A.M., Liang X., Sharma H., Lavado A. (2024). Antibody-displaying extracellular vesicles for targeted cancer therapy. Nat. Biomed. Eng..

[120] Danilushkina A.A., Emene C.C., Barlev N.A., Gomzikova M.O. (2023). Strategies for Engineering of Extracellular Vesicles. Int. J. Mol. Sci..

[121] Guo W., Shu Q., Gao L., Gao N., Wang Z., Wei W., Zhang Y., Huyan T., Li Q. (2024). A bibliometric analysis of extracellular vesicles as drug delivery vehicles in disease treatment (2010–2024). Extracell. Vesicle.

[122] Wang Z., Rich J., Hao N., Gu Y., Chen C., Yang S., Zhang P., Huang T.J. (2022). Acoustofluidics for simultaneous nanoparticle-based drug loading and exosome encapsulation. Microsyst. Nanoeng..

[123] Abas B.I., Demirbolat G.M., Cevik O. (2022). Wharton jelly-derived mesenchymal stem cell exosomes induce apoptosis and suppress EMT signaling in cervical cancer cells as an effective drug carrier system of paclitaxel. PLoS ONE.

[124] Haney M.J., Klyachko N.L., Harrison E.B., Zhao Y., Kabanov A.V., Batrakova E.V. (2019). TPP1 Delivery to Lysosomes with Extracellular Vesicles and their Enhanced Brain Distribution in the Animal Model of Batten Disease. Adv. Healthc. Mater..

[125] Yerneni S.S., Yalcintas E.P., Smith J.D., Averick S., Campbell P.G., Ozdoganlar O.B. (2022). Skin-targeted delivery of extracellular vesicle-encapsulated curcumin using dissolvable microneedle arrays. Acta Biomater..

[126] Esmaeilzadeh Gharehdaghi E., Amani A., Khoshayand M.R., Banan M., Esmaeilzadeh Gharehdaghi E., Amini M.A., Faramarzi M.A. (2014). Chitosan nanoparticles for siRNA delivery: Optimization of processing/formulation parameters. Nucleic Acid. Ther..

[127] Haney M.J., Klyachko N.L., Zhao Y., Gupta R., Plotnikova E.G., He Z., Patel T., Piroyan A., Sokolsky M., Kabanov A.V. (2015). Exosomes as drug delivery vehicles for Parkinson’s disease therapy. J. Control. Release.

[128] Liang S., Xu H., Ye B.C. (2022). Membrane-Decorated Exosomes for Combination Drug Delivery and Improved Glioma Therapy. Langmuir.

[129] Zhou S., Hu T., Zhang F., Tang D., Li D., Cao J., Wei W., Wu Y., Liu S. (2020). Integrated Microfluidic Device for Accurate Extracellular Vesicle Quantification and Protein Markers Analysis Directly from Human Whole Blood. Anal. Chem..

[130] Han B.H., Kim S., Seo G., Heo Y., Chung S., Kang J.Y. (2020). Isolation of extracellular vesicles from small volumes of plasma using a microfluidic aqueous two-phase system. Lab Chip.

[131] Wiklander O.P., Nordin J.Z., O’Loughlin A., Gustafsson Y., Corso G., Mager I., Vader P., Lee Y., Sork H., Seow Y. (2015). Extracellular vesicle in vivo biodistribution is determined by cell source, route of administration and targeting. J. Extracell. Vesicles.

[132] Banks W.A., Sharma P., Bullock K.M., Hansen K.M., Ludwig N., Whiteside T.L. (2020). Transport of Extracellular Vesicles across the Blood-Brain Barrier: Brain Pharmacokinetics and Effects of Inflammation. Int. J. Mol. Sci..

[133] Luo H., Chen D., Li R., Li R., Teng Y., Cao Y., Zou X., Wang W., Zhou C. (2023). Genetically engineered CXCR4-modified exosomes for delivery of miR-126 mimics to macrophages alleviate periodontitis. J. Nanobiotechnol..

[134] Mukerjee N., Maitra S., Kaur M., Rekha M.M., Soothwal P., Arora I., Thorat N.D., Sharma P.K., Kaushik A. (2025). Click chemistry-based modified exosomes: Towards enhancing precision in cancer theranostics. Chem. Eng. J..

[135] Song S., Shim M.K., Lim S., Moon Y., Yang S., Kim J., Hong Y., Yoon H.Y., Kim I.S., Hwang K.Y. (2020). In Situ One-Step Fluorescence Labeling Strategy of Exosomes via Bioorthogonal Click Chemistry for Real-Time Exosome Tracking In Vitro and In Vivo. Bioconjug. Chem..

[136] Zhang L., Li P., Li D., Guo S., Wang E. (2008). Effect of freeze-thawing on lipid bilayer-protected gold nanoparticles. Langmuir.

[137] Missirlis D., Krogstad D.V., Tirrell M. (2010). Internalization of p53(14–29) peptide amphiphiles and subsequent endosomal disruption results in SJSA-1 cell death. Mol. Pharm..

[138] Rubel D., Boulanger J., Craciun F., Xu E.Y., Zhang Y., Phillips L., Callahan M., Weber W., Song W., Ngai N. (2022). Anti-microRNA-21 Therapy on Top of ACE Inhibition Delays Renal Failure in Alport Syndrome Mouse Models. Cells.

[139] Mendell J.R., Rodino-Klapac L.R., Sahenk Z., Roush K., Bird L., Lowes L.P., Alfano L., Gomez A.M., Lewis S., Kota J. (2013). Eteplirsen for the treatment of Duchenne muscular dystrophy. Ann. Neurol..

[140] Parums D.V. (2024). Editorial: First Regulatory Approvals for CRISPR-Cas9 Therapeutic Gene Editing for Sickle Cell Disease and Transfusion-Dependent beta-Thalassemia. Med. Sci. Monit..

[141] U.S. Food & Drug Administration (2018). FDA Approves First-of-Its Kind Targeted RNA-Based Therapy to Treat a Rare Disease. https://www.fda.gov/news-events/press-announcements/fda-approves-first-its-kind-targeted-rna-based-therapy-treat-rare-disease.

[142] Wang Q., Sun Y., Zhang Z., Duan Y. (2015). Targeted polymeric therapeutic nanoparticles: Design and interactions with hepatocellular carcinoma. Biomaterials.

[143] Guan J., Shen Q., Zhang Z., Jiang Z., Yang Y., Lou M., Qian J., Lu W., Zhan C. (2018). Enhanced immunocompatibility of ligand-targeted liposomes by attenuating natural IgM absorption. Nat. Commun..

[144] Tzeng A., Kwan B.H., Opel C.F., Navaratna T., Wittrup K.D. (2015). Antigen specificity can be irrelevant to immunocytokine efficacy and biodistribution. Proc. Natl. Acad. Sci. USA.

[145] Mahati S., Fu X., Ma X., Zhang H., Xiao L. (2021). Delivery of miR-26a Using an Exosomes-Based Nanosystem Inhibited Proliferation of Hepatocellular Carcinoma. Front. Mol. Biosci..

[146] Izco M., Schleef M., Schmeer M., Carlos E., Verona G., Alvarez-Erviti L. (2023). Targeted Extracellular Vesicle Gene Therapy for Modulating Alpha-Synuclein Expression in Gut and Spinal Cord. Pharmaceutics.

[147] Zhang R., Fu Y., Cheng M., Ma W., Zheng N., Wang Y., Wu Z. (2022). sEVs(RVG) selectively delivers antiviral siRNA to fetus brain, inhibits ZIKV infection and mitigates ZIKV-induced microcephaly in mouse model. Mol. Ther..

[148] Yang Z., Ji P., Li Z., Zhang R., Wei M., Yang Y., Yuan L., Han Y., Yang G. (2023). Improved extracellular vesicle-based mRNA delivery for familial hypercholesterolemia treatment. Theranostics.

[149] Shrivastava S., Ray R.M., Holguin L., Echavarria L., Grepo N., Scott T.A., Burnett J., Morris K.V. (2021). Exosome-mediated stable epigenetic repression of HIV-1. Nat. Commun..

[150] Kim S.M., Yang Y., Oh S.J., Hong Y., Seo M., Jang M. (2017). Cancer-derived exosomes as a delivery platform of CRISPR/Cas9 confer cancer cell tropism-dependent targeting. J. Control. Release.

[151] Yu Y., Li W., Mao L., Peng W., Long D., Li D., Zhou R., Dang X. (2021). Genetically engineered exosomes display RVG peptide and selectively enrich a neprilysin variant: A potential formulation for the treatment of Alzheimer’s disease. J. Drug Target..

[152] Morse M.A., Garst J., Osada T., Khan S., Hobeika A., Clay T.M., Valente N., Shreeniwas R., Sutton M.A., Delcayre A. (2005). A phase I study of dexosome immunotherapy in patients with advanced non-small cell lung cancer. J. Transl. Med..

[153] Xu X., Liang Y., Li X., Ouyang K., Wang M., Cao T., Li W., Liu J., Xiong J., Li B. (2021). Exosome-mediated delivery of kartogenin for chondrogenesis of synovial fluid-derived mesenchymal stem cells and cartilage regeneration. Biomaterials.

[154] Escudier B., Dorval T., Chaput N., Andre F., Caby M.P., Novault S., Flament C., Leboulaire C., Borg C., Amigorena S. (2005). Vaccination of metastatic melanoma patients with autologous dendritic cell (DC) derived-exosomes: Results of thefirst phase I clinical trial. J. Transl. Med..

[155] Vansteenkiste J.F., Cho B.C., Vanakesa T., De Pas T., Zielinski M., Kim M.S., Jassem J., Yoshimura M., Dahabreh J., Nakayama H. (2016). Efficacy of the MAGE-A3 cancer immunotherapeutic as adjuvant therapy in patients with resected MAGE-A3-positive non-small-cell lung cancer (MAGRIT): A randomised, double-blind, placebo-controlled, phase 3 trial. Lancet Oncol..

[156] Zhang B., Ren Z., Zhao J., Zhu Y., Huang B., Xiao C., Zhang Y., Deng J., Mao L., Tang L. (2023). Global analysis of HLA-A2 restricted MAGE-A3 tumor antigen epitopes and corresponding TCRs in non-small cell lung cancer. Theranostics.

[157] Besse B., Charrier M., Lapierre V., Dansin E., Lantz O., Planchard D., Le Chevalier T., Livartoski A., Barlesi F., Laplanche A. (2016). Dendritic cell-derived exosomes as maintenance immunotherapy after first line chemotherapy in NSCLC. Oncoimmunology.

[158] (2020). A First-in-Human Study of CDK-002 (exoSTING) in Subjects with Advanced/Metastatic, Recurrent, Injectable Solid Tumors, with Emphasis on Squamous Cell Carcinoma of the Head and Neck, Triple Negative Breast Cancer, Anaplastic Thyroid Carcinoma, and Cutaneous Squamous Cell Carcinoma. https://clinicaltrials.gov/study/NCT04592484.

[159] (2018). Phase I Study of Mesenchymal Stromal Cells-Derived Exosomes with KrasG12D SiRNA for Metastatic Pancreas Cancer Patients Harboring KrasG12D Mutation. https://clinicaltrials.gov/study/NCT03608631.

[160] (2012). Phase 1 Study in Humans Evaluating the Safety of Rectus Sheath Implantation of Diffusion Chambers Encapsulating Autologous Malignant Glioma Cells Treated With Insulin-Like Growth Factor Receptor-1 Antisense Oligodeoxynucleotide in 12 Patients with Recurrent Malignant Glioma. https://clinicaltrials.gov/study/NCT01550523.

[161] Maris C., D’Haene N., Trepant A.L., Le Mercier M., Sauvage S., Allard J., Rorive S., Demetter P., Decaestecker C., Salmon I. (2015). IGF-IR: A new prognostic biomarker for human glioblastoma. Br. J. Cancer.

[162] Judy K.D., Andrews D.W., Harshyne L., Kenyon L., Talekar K., Atsina K.-B., Kim L., Shi W., Werner-Wasik M., Kean R. (2020). Abstract B71: Phase 1b/2 prospective randomized trial of four autologous cell vaccine dose cohorts for initial treatment of glioblastoma. Cancer Immunol. Res..

[163] James Graham Brown Cancer Center (2012). Preliminary Clinical Trial Investigating the Ability of Plant Exosomes to Abrogate Oral Mucositis Induced by Combined Chemotherapy and Radiation in Head and Neck Cancer Patients. https://clinicaltrials.gov/study/NCT01668849.

[164] Farina E., Daghero H., Bollati-Fogolin M., Boido E., Cantero J., Moncada-Basualto M., Olea-Azar C., Polticelli F., Paulino M. (2023). Antioxidant Capacity and NF-kB-Mediated Anti-Inflammatory Activity of Six Red Uruguayan Grape Pomaces. Molecules.

[165] Mandic A.I., Đilas S.M., Ćetković G.S., Čanadanović-Brunet J.M., Tumbas V.T. (2008). Polyphenolic Composition and Antioxidant Activities of Grape Seed Extract. Int. J. Food Prop..

[166] Grigoropoulos I., Tsioulos G., Kastrissianakis A., Shapira S., Green O., Rapti V., Tsakona M., Konstantinos T., Savva A., Kavatha D. (2024). The safety and potential efficacy of exosomes overexpressing CD24 (EXO-CD24) in mild-moderate COVID-19 related ARDS. Respir. Res..

[167] (2011). Phase I Clinical Trial Investigating the Ability of Plant Exosomes to Deliver Curcumin to Normal and Malignant Colon Tissue. https://clinicaltrials.gov/study/NCT01294072.

[168] Aegle Therapeutics Corp (2025). Dystrophic Epidermolysis Bullosa. https://aegletherapeutics.com/pipeline/.

[169] BioSpace. Aegle Therapeutics Corp (2024). Announces Positive Data for the First Patient in a Phase 1/2a Clinical Trial Dosed With AGLE-102™, a Novel Extracellular Vesicle Therapy. https://www.biospace.com/aegle-therapeutics-corp-announces-positive-data-for-the-first-patient-in-a-phase-1-2a-clinical-trial-dosed-with-agle-102-a-novel-extracellular-vesicle-therapy.

[170] Tan S.T., Aisyah P.B., Firmansyah Y., Nathasia N., Budi E., Hendrawan S. (2023). Effectiveness of Secretome from Human Umbilical Cord Mesenchymal Stem Cells in Gel (10% SM-hUCMSC Gel) for Chronic Wounds (Diabetic and Trophic Ulcer). J. Multidiscip. Healthc..

[171] Air Force Military Medical University, China (2021). Exosome-Based Nanoplatform for Ldlr mRNA Delivery in Familial Hypercholesterolemia. https://clinicaltrials.gov/study/NCT05043181.

[172] Harshyne L.A., Hooper K.M., Andrews E.G., Nasca B.J., Kenyon L.C., Andrews D.W., Hooper D.C. (2015). Glioblastoma exosomes and IGF-1R/AS-ODN are immunogenic stimuli in a translational research immunotherapy paradigm. Cancer Immunol. Immunother..

[173] (2010). Phase II Trial of a Vaccination with Tumor Antigen-Loaded Dendritic Cell-derived Exosomes on Patients with Unresectable Non Small Cell Lung Cancer Responding to Induction Chemotherapy. https://clinicaltrials.gov/study/NCT01159288.

[174] Congressionally Directed Medical Research Programs (2021). A Pilot Safety Study of Mesenchymal Stem Cell Derived Extracellular Vesicles for the Treatment of Burn Wounds. https://clinicaltrials.gov/study/NCT05078385.

[175] Stem Cell and Cancer Institute, Kalbe Farma Tbk, PT Pharma Metric Labs (2019). Therapeutic Potential of Stem Cell Conditioned Medium on Chronic Ulcer Wounds: Pilot Study in Human. https://clinicaltrials.gov/study/NCT04134676.

[176] Costa M.H.G., Costa M.S., Painho B., Sousa C.D., Carrondo I., Oltra E., Pelacho B., Prosper F., Isidro I.A., Alves P. (2023). Enhanced bioprocess control to advance the manufacture of mesenchymal stromal cell-derived extracellular vesicles in stirred-tank bioreactors. Biotechnol. Bioeng..

[177] Visan K.S., Lobb R.J., Ham S., Lima L.G., Palma C., Edna C.P.Z., Wu L.Y., Gowda H., Datta K.K., Hartel G. (2022). Comparative analysis of tangential flow filtration and ultracentrifugation, both combined with subsequent size exclusion chromatography, for the isolation of small extracellular vesicles. J. Extracell. Vesicles.

[178] Busatto S., Vilanilam G., Ticer T., Lin W.L., Dickson D.W., Shapiro S., Bergese P., Wolfram J. (2018). Tangential Flow Filtration for Highly Efficient Concentration of Extracellular Vesicles from Large Volumes of Fluid. Cells.

[179] Dobnik D., Kogovsek P., Jakomin T., Kosir N., Tusek Znidaric M., Leskovec M., Kaminsky S.M., Mostrom J., Lee H., Ravnikar M. (2019). Accurate Quantification and Characterization of Adeno-Associated Viral Vectors. Front. Microbiol..

[180] Man K., Barroso I.A., Brunet M.Y., Peacock B., Federici A.S., Hoey D.A., Cox S.C. (2022). Controlled Release of Epigenetically-Enhanced Extracellular Vesicles from a GelMA/Nanoclay Composite Hydrogel to Promote Bone Repair. Int. J. Mol. Sci..

[181] Patel N., Avery E., Chung E.J. (2024). Supramolecular hydrogels for sustained extracellular vesicle delivery. MRS Commun..

[182] Zhang W., Yang G., Zhang A., Xu L.X., He X. (2010). Preferential vitrification of water in small alginate microcapsules significantly augments cell cryopreservation by vitrification. Biomed. Microdevices.

[183] Peinado H., Aleckovic M., Lavotshkin S., Matei I., Costa-Silva B., Moreno-Bueno G., Hergueta-Redondo M., Williams C., Garcia-Santos G., Ghajar C. (2012). Melanoma exosomes educate bone marrow progenitor cells toward a pro-metastatic phenotype through MET. Nat. Med..

[184] Umezu T., Tadokoro H., Azuma K., Yoshizawa S., Ohyashiki K., Ohyashiki J.H. (2014). Exosomal miR-135b shed from hypoxic multiple myeloma cells enhances angiogenesis by targeting factor-inhibiting HIF-1. Blood.

[185] Munagala R., Aqil F., Jeyabalan J., Kandimalla R., Wallen M., Tyagi N., Wilcher S., Yan J., Schultz D.J., Spencer W. (2021). Exosome-mediated delivery of RNA and DNA for gene therapy. Cancer Lett..

[186] Ma J., Zhang S., Liu J., Liu F., Du F., Li M., Chen A.T., Bao Y., Suh H.W., Avery J. (2019). Targeted Drug Delivery to Stroke via Chemotactic Recruitment of Nanoparticles Coated with Membrane of Engineered Neural Stem Cells. Small.

[187] Molinaro R., Corbo C., Martinez J.O., Taraballi F., Evangelopoulos M., Minardi S., Yazdi I.K., Zhao P., De Rosa E., Sherman M.B. (2016). Biomimetic proteolipid vesicles for targeting inflamed tissues. Nat. Mater..

